# Supplementary material for: Interaction of high-fat diet and brain trauma alters adipose tissue macrophages and brain microglia associated with exacerbated cognitive dysfunction
Source: J Neuroinflammation. 2024 Apr 29;21:113. doi: 10.1186/s12974-024-03107-6 (PMC11058055; doi:10.1186/s12974-024-03107-6)
Supplement: Supplementary file 1 — Additional file 1: Figure S1. Gating strategy for CD45+/CD11b+ and CD11b+ Red Bead+ ATM/myeloid cell populations. A Gating strategy for CD45+/CD11b+ ATM/myeloid cell populations in the adipose tissue at 28 dpi. B Gating strategy for CD11b+ Red Bead+ ATM/myeloid cell population in the adipose tissue at 28 dpi. Although there was a factor effect of diet on % Red Bead+ of CD11b+ ATM/myeloid cells, there was no reported differences between groups in post-hoc analysis (C). Post-hoc analysis revealed that TBI-HFD had a significant increase in MFI of Red Bead+ of CD11b+ ATM/myeloid cells when compared to TBI-SD counterparts (D). Figure S2. Morpheus unsupervised hierarchical clustering heat map on VAT cellular transcriptome. Only genes that showed evidence of significant changes (p < 0.05 in at least one of the two-way ANOVA outcomes) were included (369 genes). The heat map demonstrated the same ordered clustering across the groups observed Fig. 9A. Figure S3. IPA detects Upstream Regulator-Genes upregulated and downregulated genes in VAT. IPA determined the activation of pathways based on the inclusion of genes with p < 0.05; all pathways with z-score > 2.8 or < − 2.8 in at least one group are presented. Analysis of the Z-score of adipose upstream upregulated genes pathways including multiple inflammatory-related, identify significant TBI, HFD, and TBI-HFD interaction effects. Notably, TBI-HFD significantly increases upregulated pathways compared to TBI-SD. Only diet was a significant factor for downregulated genes. Figure S4. IPA detects activated and inhibited Upstream Regulator-Drugs pathways in VAT. IPA determined the activation of pathways based on the inclusion of genes with p < 0.05; all pathways with z-score > 2.8 or < − 2.8 in at least one group are presented. Analysis of the Z-score of adipose upstream upregulated and downregulated drugs pathways, including multiple inflammatory-related, identify significant TBI, HFD, and TBI-HFD interaction effects. Notably, TBI [file 12974_2024_3107_MOESM1_ESM.pdf]

Figure 1-Supplemental

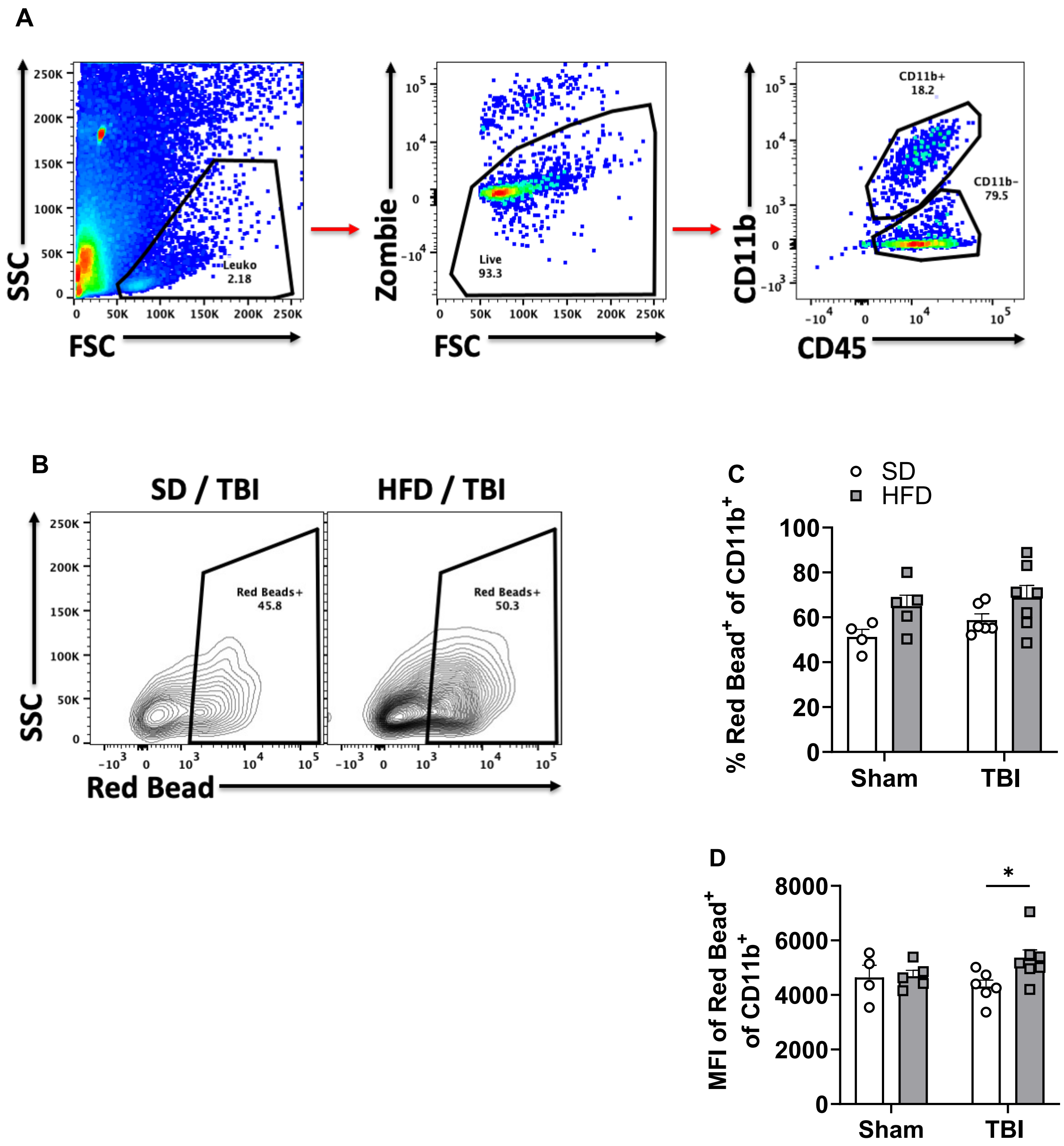

Figure 2-Supplemental

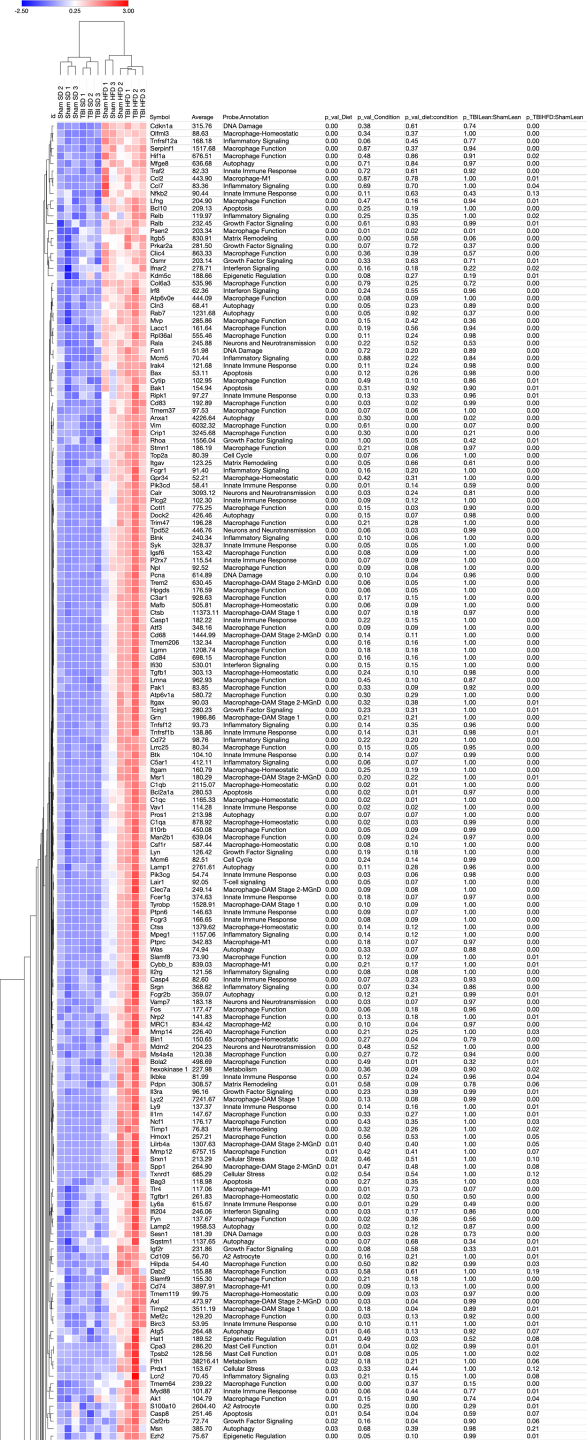

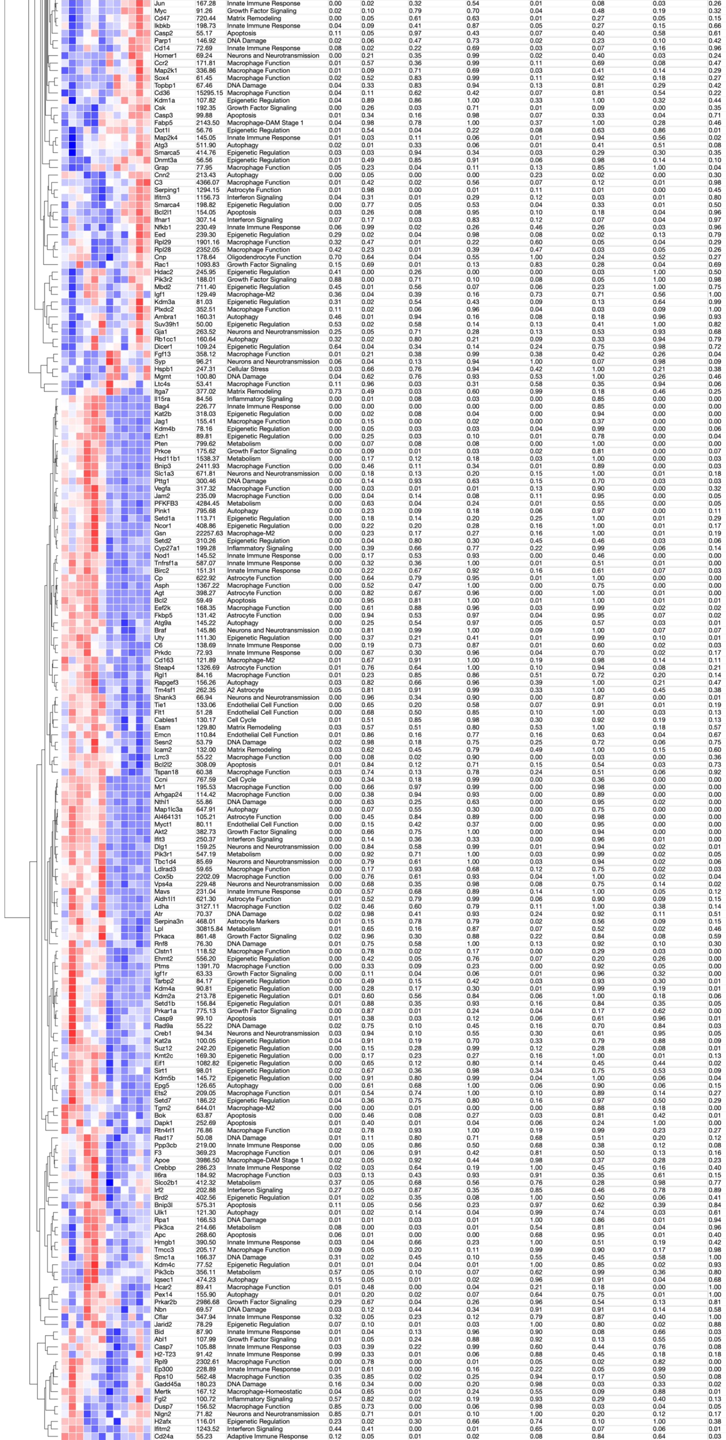

Symbol	Average	Probe.Annotation	p_val_Diet	p_val_Condition	p_val_diet:condition	p_TBILean:ShamLean	p_TBIHFD:ShamLean	p_TBIHFD:ShamHFD	p_TBIHFD:TBILean	p_ShamHFD:ShamLean
Cdkn1a	315.76	DNA Damage	0.00	0.38	0.61	0.74	0.00	0.99	0.00	0.00
Olfml3	88.63	Macrophage-Homeostatic	0.00	0.34	0.37	1.00	0.00	0.53	0.00	0.00
Tnfrsf12a	168.18	Inflammatory Signaling	0.00	0.06	0.45	0.77	0.00	0.24	0.00	0.00
Serpinf1	1517.68	Macrophage Function	0.00	0.87	0.37	0.94	0.00	0.86	0.00	0.00
Hif1a	676.51	Macrophage Function	0.00	0.48	0.86	0.91	0.02	0.98	0.01	0.01
Mfge8	636.68	Autophagy	0.00	0.71	0.84	0.97	0.00	1.00	0.00	0.00
Traf2	82.33	Innate Immune Response	0.00	0.72	0.61	0.92	0.00	1.00	0.00	0.00
Ccl2	443.90	Macrophage-M1	0.00	0.87	0.78	1.00	0.01	0.99	0.01	0.01
Ccl7	83.36	Inflammatory Signaling	0.00	0.69	0.70	1.00	0.04	0.94	0.04	0.02
Nfkb2	90.44	Innate Immune Response	0.00	0.11	0.63	0.43	0.13	0.80	0.01	0.04
Lfng	204.90	Macrophage Function	0.00	0.47	0.16	0.94	0.01	0.41	0.00	0.05
Bcl10	209.13	Apoptosis	0.00	0.25	0.19	1.00	0.00	0.31	0.00	0.05
Relb	119.97	Inflammatory Signaling	0.00	0.25	0.35	1.00	0.02	0.44	0.02	0.14
Ralb	232.45	Growth Factor Signaling	0.00	0.61	0.93	0.99	0.01	0.97	0.02	0.02
Psen2	203.34	Macrophage Function	0.00	0.01	0.02	0.01	0.00	1.00	0.03	0.00
Itgb5	830.91	Matrix Remodeling	0.00	0.00	0.58	0.06	0.00	0.02	0.00	0.00
Prkar2a	281.50	Growth Factor Signaling	0.00	0.07	0.72	0.37	0.00	0.65	0.02	0.01
Clic4	863.33	Macrophage Function	0.00	0.36	0.39	0.57	0.00	1.00	0.02	0.00
Osmr	203.14	Growth Factor Signaling	0.00	0.33	0.63	0.71	0.01	0.98	0.05	0.02
Ifnar2	278.71	Interferon Signaling	0.00	0.16	0.18	0.22	0.02	1.00	0.36	0.02
Kdm5c	188.66	Epigenetic Regulation	0.00	0.08	0.27	0.19	0.01	0.93	0.25	0.03
Col6a3	535.96	Macrophage Function	0.00	0.79	0.25	0.72	0.00	0.90	0.00	0.00
Irf8	62.36	Interferon Signaling	0.00	0.24	0.55	0.96	0.00	0.57	0.00	0.00
Atp6v0e	444.09	Macrophage Function	0.00	0.08	0.09	1.00	0.00	0.09	0.00	0.00
Cln3	68.41	Autophagy	0.00	0.05	0.23	0.89	0.00	0.13	0.00	0.00
Rab7	1231.68	Autophagy	0.00	0.05	0.92	0.37	0.00	0.44	0.00	0.00
Mvp	285.86	Macrophage Function	0.00	0.15	0.42	0.36	0.00	0.95	0.01	0.00
Lacc1	161.64	Macrophage Function	0.00	0.19	0.56	0.94	0.00	0.52	0.00	0.00
Rpl36al	555.46	Macrophage Function	0.00	0.11	0.24	0.98	0.00	0.21	0.00	0.00
Rala	245.88	Neurons and Neurotransmission	0.00	0.22	0.52	0.53	0.00	0.97	0.00	0.00
Fen1	51.98	DNA Damage	0.00	0.72	0.20	0.89	0.00	0.62	0.00	0.01
Mcm5	70.44	Inflammatory Signaling	0.00	0.88	0.22	0.84	0.00	0.73	0.00	0.01
Irak4	121.68	Innate Immune Response	0.00	0.11	0.24	0.98	0.00	0.20	0.00	0.00
Bax	53.11	Apoptosis	0.00	0.12	0.26	0.98	0.00	0.23	0.00	0.02
Cytip	102.95	Macrophage Function	0.00	0.49	0.10	0.86	0.01	0.33	0.00	0.08
Bak1	154.94	Apoptosis	0.00	0.31	0.92	0.90	0.01	0.84	0.04	0.04
Ripk1	97.27	Innate Immune Response	0.00	0.13	0.33	0.96	0.01	0.29	0.02	0.12
Cd83	192.89	Macrophage Function	0.00	0.03	0.02	0.99	0.00	0.02	0.00	0.00
Tmem37	97.53	Macrophage Function	0.00	0.07	0.06	1.00	0.00	0.06	0.00	0.00
Anxa1	4226.64	Autophagy	0.00	0.30	0.00	0.02	0.00	0.00	0.00	0.00
Vim	6032.32	Macrophage Function	0.00	0.61	0.00	0.07	0.00	0.03	0.00	0.00
Crip1	3245.68	Macrophage Function	0.00	0.30	0.00	0.21	0.00	0.02	0.00	0.01
Rhoa	1556.04	Growth Factor Signaling	0.00	1.00	0.05	0.42	0.01	0.42	0.00	0.08
Stmn1	186.19	Macrophage Function	0.00	0.21	0.08	0.97	0.00	0.16	0.00	0.00
Top2a	80.39	Cell Cycle	0.00	0.07	0.06	1.00	0.00	0.07	0.00	0.00
Itgav	123.25	Matrix Remodeling	0.00	0.05	0.66	0.61	0.00	0.29	0.00	0.01
Fcgr1	91.40	Inflammatory Signaling	0.00	0.16	0.20	1.00	0.00	0.24	0.00	0.04
Gpr34	52.21	Macrophage-Homeostatic	0.00	0.42	0.31	1.00	0.00	0.55	0.00	0.03
Pik3cd	58.41	Innate Immune Response	0.00	0.01	0.14	0.59	0.00	0.03	0.00	0.00
Calr	3093.12	Neurons and Neurotransmission	0.00	0.03	0.24	0.81	0.00	0.10	0.00	0.00
Plcg2	102.30	Innate Immune Response	0.00	0.09	0.12	1.00	0.00	0.12	0.00	0.01
Cotl1	775.25	Macrophage Function	0.00	0.15	0.03	0.90	0.00	0.07	0.00	0.01
Dock2	426.46	Autophagy	0.00	0.15	0.07	0.98	0.00	0.11	0.00	0.01
Trim47	196.28	Macrophage Function	0.00	0.21	0.28	1.00	0.00	0.35	0.00	0.01
Tpd52	446.76	Neurons and Neurotransmission	0.00	0.06	0.03	0.99	0.00	0.04	0.00	0.00
Blnk	240.34	Inflammatory Signaling	0.00	0.10	0.06	1.00	0.00	0.08	0.00	0.00
Syk	328.37	Innate Immune Response	0.00	0.05	0.05	1.00	0.00	0.05	0.00	0.00
Igsf6	153.42	Macrophage Function	0.00	0.08	0.09	1.00	0.00	0.09	0.00	0.01
P2rx7	115.54	Innate Immune Response	0.00	0.07	0.09	1.00	0.00	0.08	0.00	0.00
Npl	92.52	Macrophage Function	0.00	0.09	0.08	1.00	0.00	0.09	0.00	0.01
Pcna	614.89	DNA Damage	0.00	0.10	0.04	0.96	0.00	0.06	0.00	0.01
Trem2	630.45	Macrophage-DAM Stage 2-MGnD	0.00	0.06	0.05	1.00	0.00	0.05	0.00	0.01
Hpgds	176.59	Macrophage Function	0.00	0.06	0.05	1.00	0.00	0.05	0.00	0.02
C3ar1	928.63	Macrophage Function	0.00	0.17	0.15	1.00	0.00	0.20	0.00	0.01
Mafb	505.81	Macrophage-Homeostatic	0.00	0.06	0.09	1.00	0.00	0.07	0.00	0.01
Ctsb	11373.11	Macrophage-DAM Stage 1	0.00	0.07	0.18	0.97	0.00	0.13	0.00	0.01
Casp1	182.22	Innate Immune Response	0.00	0.22	0.15	1.00	0.00	0.25	0.00	0.01
Atf3	348.16	Macrophage Function	0.00	0.09	0.09	1.00	0.00	0.09	0.00	0.01
Cd68	1444.99	Macrophage-DAM Stage 2-MGnD	0.00	0.14	0.11	1.00	0.00	0.15	0.00	0.01
Tmem206	132.34	Macrophage Function	0.00	0.16	0.16	1.00	0.00	0.21	0.00	0.01
Lgmn	1208.74	Macrophage Function	0.00	0.18	0.18	1.00	0.00	0.24	0.00	0.01
Cd84	698.15	Macrophage Function	0.00	0.16	0.16	1.00	0.00	0.21	0.00	0.02
Ifi30	530.01	Interferon Signaling	0.00	0.15	0.15	1.00	0.00	0.19	0.00	0.02
Tgfb1	303.13	Macrophage-Homeostatic	0.00	0.24	0.10	0.98	0.00	0.21	0.00	0.02
Lmna	962.93	Macrophage Function	0.00	0.45	0.10	0.87	0.00	0.31	0.00	0.01
Pak1	83.85	Macrophage Function	0.00	0.33	0.09	0.92	0.00	0.23	0.00	0.03
Atp6v1a	580.72	Macrophage Function	0.00	0.30	0.29	1.00	0.00	0.44	0.00	0.03
Itgax	90.03	Macrophage-DAM Stage 2-MGnD	0.00	0.32	0.38	1.00	0.01	0.53	0.01	0.04
Tcirg1	280.23	Growth Factor Signaling	0.00	0.23	0.31	1.00	0.01	0.39	0.01	0.05
Grn	1986.86	Macrophage-DAM Stage 1	0.00	0.21	0.21	1.00	0.00	0.30	0.00	0.05
Tnfsf12	93.73	Inflammatory Signaling	0.00	0.14	0.35	0.96	0.00	0.31	0.00	0.03
Tnfrsf1b	138.86	Innate Immune Response	0.00	0.14	0.31	0.98	0.01	0.29	0.01	0.07
Cd72	98.76	Inflammatory Signaling	0.00	0.22	0.20	1.00	0.00	0.29	0.00	0.02
Lrrc25	80.34	Macrophage Function	0.00	0.15	0.05	0.95	0.00	0.09	0.00	0.01
Btk	104.10	Innate Immune Response	0.00	0.14	0.07	0.99	0.00	0.11	0.00	0.04
C5ar1	412.11	Inflammatory Signaling	0.00	0.06	0.07	1.00	0.00	0.07	0.00	0.03
Itgam	160.79	Macrophage-Homeostatic	0.00	0.25	0.19	1.00	0.00	0.30	0.00	0.05
Msr1	180.29	Macrophage-DAM Stage 2-MGnD	0.00	0.20	0.22	1.00	0.01	0.29	0.01	0.07
C1qb	2115.07	Macrophage-Homeostatic	0.00	0.02	0.01	1.00	0.00	0.01	0.00	0.00
Bcl2a1a	280.53	Apoptosis	0.00	0.02	0.01	0.97	0.00	0.01	0.00	0.01
C1qc	1165.33	Macrophage-Homeostatic	0.00	0.02	0.01	1.00	0.00	0.01	0.00	0.01
Vav1	114.28	Innate Immune Response	0.00	0.02	0.02	1.00	0.00	0.02	0.00	0.03
Pros1	213.98	Autophagy	0.00	0.07	0.07	1.00	0.00	0.07	0.00	0.03
C1qa	878.92	Macrophage-Homeostatic	0.00	0.02	0.03	0.99	0.00	0.02	0.00	0.02
Il10rb	450.08	Macrophage Function	0.00	0.05	0.09	0.99	0.00	0.07	0.00	0.04
Man2b1	639.04	Macrophage Function	0.00	0.09	0.24	0.97	0.00	0.19	0.00	0.01
Csf1r	587.44	Macrophage-Homeostatic	0.00	0.08	0.10	1.00	0.00	0.10	0.00	0.05
Lyn	126.42	Growth Factor Signaling	0.00	0.19	0.18	1.00	0.00	0.25	0.00	0.04
Mcm6	82.51	Cell Cycle	0.00	0.24	0.14	0.99	0.00	0.24	0.00	0.05
Lamp1	2761.61	Autophagy	0.00	0.11	0.28	0.96	0.00	0.24	0.00	0.02
Pik3cg	54.74	Innate Immune Response	0.00	0.03	0.06	0.98	0.00	0.03	0.00	0.04
Lair1	92.05	T-cell signaling	0.00	0.05	0.07	1.00	0.00	0.06	0.00	0.06
Clec7a	249.14	Macrophage-DAM Stage 2-MGnD	0.00	0.09	0.08	1.00	0.00	0.09	0.00	0.12
Fcer1g	374.63	Innate Immune Response	0.00	0.18	0.07	0.97	0.00	0.14	0.00	0.05
Tyrobp	1528.91	Macrophage-DAM Stage 1	0.00	0.10	0.09	1.00	0.00	0.11	0.00	0.05
Ptpn6	146.63	Innate Immune Response	0.00	0.09	0.07	1.00	0.00	0.09	0.00	0.04
Fcgr3	166.65	Innate Immune Response	0.00	0.08	0.09	1.00	0.00	0.09	0.00	0.05
Ctss	1379.62	Macrophage-Homeostatic	0.00	0.14	0.12	1.00	0.00	0.15	0.00	0.05
Mpeg1	1157.06	Inflammatory Signaling	0.00	0.14	0.12	1.00	0.00	0.16	0.00	0.07
Ptprc	342.83	Macrophage-M1	0.00	0.18	0.07	0.97	0.00	0.14	0.00	0.09
Was	74.94	Autophagy	0.00	0.33	0.07	0.88	0.00	0.21	0.00	0.09
Slamf8	73.90	Macrophage Function	0.00	0.12	0.09	1.00	0.01	0.12	0.00	0.16
Cybb_b	839.03	Macrophage-M1	0.00	0.21	0.17	1.00	0.01	0.25	0.01	0.21
Il2rg	121.56	Inflammatory Signaling	0.00	0.08	0.08	1.00	0.00	0.08	0.00	0.20
Casp4	82.60	Innate Immune Response	0.00	0.07	0.23	0.93	0.00	0.15	0.00	0.05
Srgn	368.62	Inflammatory Signaling	0.00	0.07	0.34	0.86	0.00	0.20	0.01	0.06
Fcgr2b	359.07	Autophagy	0.00	0.12	0.21	0.99	0.01	0.20	0.01	0.16
Vamp7	183.18	Neurons and Neurotransmission	0.00	0.03	0.07	0.97	0.00	0.04	0.00	0.03
Fos	177.47	Macrophage Function	0.00	0.06	0.18	0.96	0.00	0.13	0.00	0.02
Nrp2	141.83	Macrophage Function	0.00	0.13	0.18	1.00	0.01	0.19	0.01	0.12
MRC1	834.42	Macrophage-M2	0.00	0.10	0.04	0.97	0.00	0.06	0.00	0.21
Mmp14	226.40	Macrophage Function	0.00	0.21	0.25	1.00	0.03	0.32	0.03	0.32
Bin1	150.65	Macrophage-Homeostatic	0.00	0.27	0.04	0.79	0.00	0.11	0.00	0.08
Mdm2	204.23	Neurons and Neurotransmission	0.00	0.48	0.52	1.00	0.00	0.76	0.00	0.01
Ms4a4a	120.38	Macrophage Function	0.00	0.27	0.72	0.94	0.00	0.70	0.01	0.01
Bola2	498.69	Macrophage Function	0.00	0.49	0.01	0.32	0.01	0.08	0.00	0.43
hexokinase 1	227.98	Metabolism	0.00	0.36	0.09	0.90	0.02	0.24	0.01	0.41
Ikbke	81.99	Innate Immune Response	0.00	0.57	0.24	0.96	0.04	0.58	0.02	0.23
Pdpn	308.57	Matrix Remodeling	0.01	0.58	0.09	0.78	0.06	0.35	0.02	0.56
Il3ra	96.16	Growth Factor Signaling	0.00	0.23	0.39	0.99	0.01	0.45	0.01	0.08
Lyz2	7241.67	Macrophage-DAM Stage 1	0.00	0.13	0.08	0.99	0.00	0.12	0.00	0.15
Ly9	137.37	Innate Immune Response	0.00	0.14	0.16	1.00	0.01	0.19	0.01	0.14
Il1rn	147.67	Macrophage Function	0.00	0.33	0.27	1.00	0.01	0.44	0.01	0.10
Ncf1	176.17	Macrophage Function	0.00	0.43	0.35	1.00	0.03	0.59	0.03	0.19
Timp1	76.83	Matrix Remodeling	0.00	0.32	0.26	1.00	0.02	0.43	0.01	0.15
Hmox1	257.21	Macrophage Function	0.00	0.56	0.53	1.00	0.05	0.81	0.05	0.18
Lilrb4a	1307.63	Macrophage-DAM Stage 2-MGnD	0.01	0.40	0.40	1.00	0.05	0.61	0.05	0.31
Mmp12	6757.15	Macrophage Function	0.01	0.42	0.41	1.00	0.07	0.64	0.07	0.37
Srxn1	213.29	Cellular Stress	0.02	0.46	0.51	1.00	0.10	0.73	0.11	0.39
Spp1	264.90	Macrophage-DAM Stage 2-MGnD	0.01	0.47	0.48	1.00	0.08	0.72	0.08	0.34
Txnrd1	685.29	Cellular Stress	0.02	0.54	0.54	1.00	0.12	0.80	0.12	0.41
Bag3	118.98	Apoptosis	0.00	0.27	0.35	1.00	0.03	0.46	0.04	0.25
Tlr4	117.06	Macrophage-M1	0.00	0.01	0.73	0.07	0.00	0.15	0.01	0.01
Tgfbr1	261.83	Macrophage-Homeostatic	0.00	0.02	0.50	0.50	0.00	0.14	0.01	0.04
Ly6a	615.67	Innate Immune Response	0.00	0.01	0.29	0.49	0.00	0.06	0.01	0.06
Ifi204	246.06	Interferon Signaling	0.00	0.03	0.17	0.86	0.00	0.08	0.01	0.17
Fyn	137.67	Macrophage Function	0.00	0.02	0.36	0.56	0.00	0.10	0.01	0.09
Lamp2	1958.53	Autophagy	0.00	0.02	0.12	0.87	0.00	0.05	0.01	0.25
Sesn1	181.39	DNA Damage	0.00	0.03	0.28	0.73	0.00	0.11	0.02	0.17
Sqstm1	1137.65	Autophagy	0.00	0.07	0.68	0.34	0.01	0.65	0.13	0.06
Igf2r	231.86	Growth Factor Signaling	0.00	0.08	0.58	0.33	0.01	0.74	0.09	0.03
Cd109	56.70	A2 Astrocyte	0.00	0.16	0.21	1.00	0.01	0.24	0.01	0.20
Hilpda	54.40	Macrophage Function	0.00	0.50	0.82	0.99	0.03	0.91	0.05	0.08
Dab2	155.88	Macrophage Function	0.03	0.58	0.61	1.00	0.19	0.86	0.20	0.50
Slamf9	155.30	Macrophage Function	0.00	0.21	0.18	1.00	0.00	0.26	0.00	0.07
Cd74	3897.91	Macrophage-M1	0.00	0.09	0.13	1.00	0.00	0.12	0.00	0.10
Tmem119	99.75	Macrophage-Homeostatic	0.00	0.09	0.03	0.97	0.00	0.05	0.00	0.05
Axl	473.97	Macrophage-DAM Stage 2-MGnD	0.00	0.03	0.04	0.99	0.00	0.03	0.00	0.13
Timp2	3511.19	Macrophage-DAM Stage 1	0.00	0.18	0.04	0.89	0.01	0.09	0.00	0.48
Mef2c	129.20	Macrophage Function	0.00	0.03	0.13	0.92	0.00	0.06	0.00	0.11
Birc3	53.95	Innate Immune Response	0.00	0.10	0.11	1.00	0.01	0.12	0.01	0.30
Atg5	264.48	Autophagy	0.01	0.46	0.13	0.92	0.07	0.37	0.03	0.63
Hat1	189.52	Epigenetic Regulation	0.01	0.49	0.03	0.52	0.08	0.15	0.01	0.98
Cpa3	286.20	Mast Cell Function	0.01	0.04	0.02	0.99	0.01	0.03	0.01	0.88
Tpsb2	128.56	Mast Cell Function	0.01	0.08	0.05	1.00	0.02	0.06	0.02	0.90
Fth1	38216.41	Metabolism	0.02	0.18	0.21	1.00	0.06	0.26	0.07	0.72
Prdx1	153.67	Cellular Stress	0.03	0.33	0.44	1.00	0.12	0.58	0.15	0.61
Lcn2	70.45	Inflammatory Signaling	0.03	0.21	0.15	1.00	0.08	0.24	0.06	0.84
Tmem64	239.22	Macrophage Function	0.00	0.00	0.37	0.15	0.00	0.02	0.00	0.03
Myd88	101.87	Innate Immune Response	0.00	0.06	0.44	0.77	0.01	0.23	0.03	0.17
Ak1	104.79	Macrophage Function	0.01	0.15	0.90	0.74	0.04	0.64	0.16	0.20
S100a10	2604.40	A2 Astrocyte	0.00	0.25	0.00	0.29	0.01	0.03	0.00	0.93
Casp8	251.46	Apoptosis	0.01	0.54	0.04	0.59	0.07	0.20	0.01	0.86
Csf2rb	72.74	Growth Factor Signaling	0.02	0.16	0.04	0.90	0.06	0.08	0.02	1.00
Msn	385.70	Autophagy	0.03	0.68	0.39	0.98	0.21	0.78	0.13	0.62
Ezh2	75.67	Epigenetic Regulation	0.00	0.05	0.10	0.99	0.01	0.08	0.01	0.39
Jun	167.28	Innate Immune Response	0.00	0.02	0.32	0.54	0.01	0.08	0.03	0.26
Myc	91.26	Growth Factor Signaling	0.02	0.10	0.79	0.70	0.04	0.48	0.19	0.32
Cd47	720.44	Matrix Remodeling	0.00	0.05	0.61	0.63	0.01	0.27	0.05	0.15
Ikbkb	198.73	Innate Immune Response	0.04	0.09	0.41	0.87	0.05	0.27	0.15	0.66
Casp2	55.17	Apoptosis	0.11	0.05	0.97	0.43	0.07	0.40	0.58	0.61
Parp1	146.92	DNA Damage	0.02	0.06	0.47	0.73	0.02	0.23	0.10	0.42
Cd14	72.69	Innate Immune Response	0.08	0.02	0.22	0.69	0.03	0.07	0.16	0.96
Homer1	69.24	Neurons and Neurotransmission	0.00	0.21	0.35	0.99	0.02	0.40	0.03	0.24
Ccr2	171.81	Macrophage Function	0.01	0.57	0.36	0.99	0.11	0.69	0.08	0.47
Map2k1	336.86	Macrophage Function	0.01	0.09	0.71	0.69	0.03	0.41	0.14	0.29
Sox4	61.45	Macrophage Function	0.02	0.52	0.83	0.99	0.11	0.92	0.18	0.27
Topbp1	67.46	DNA Damage	0.04	0.33	0.83	0.94	0.13	0.81	0.29	0.42
Cd36	15295.15	Macrophage Function	0.04	0.11	0.62	0.42	0.07	0.81	0.54	0.22
Kdm1a	107.82	Epigenetic Regulation	0.04	0.89	0.86	1.00	0.33	1.00	0.32	0.44
Csk	192.35	Growth Factor Signaling	0.00	0.26	0.03	0.71	0.01	0.09	0.00	0.35
Casp3	99.88	Apoptosis	0.01	0.34	0.16	0.98	0.07	0.33	0.04	0.71
Fabp5	2143.50	Macrophage-DAM Stage 1	0.04	0.98	0.78	1.00	0.37	1.00	0.28	0.46
Dot1l	56.76	Epigenetic Regulation	0.01	0.54	0.04	0.22	0.08	0.63	0.86	0.01
Map2k4	145.05	Innate Immune Response	0.01	0.03	0.11	0.06	0.01	0.94	0.56	0.02
Atg3	511.90	Autophagy	0.02	0.01	0.33	0.06	0.01	0.41	0.51	0.08
Smarca5	414.76	Epigenetic Regulation	0.03	0.03	0.94	0.34	0.03	0.29	0.30	0.35
Dnmt3a	56.56	Epigenetic Regulation	0.01	0.49	0.85	0.91	0.06	0.98	0.14	0.10
Grap	77.95	Macrophage Function	0.05	0.23	0.04	0.11	0.13	0.85	1.00	0.04
Cnn2	213.43	Autophagy	0.00	0.05	0.00	0.00	0.23	0.02	0.00	0.30
C3	4366.07	Macrophage Function	0.01	0.42	0.02	0.56	0.07	0.12	0.01	0.98
Serping1	1294.15	Astrocyte Function	0.01	0.98	0.00	0.01	0.11	0.01	0.00	0.45
Ifitm3	1156.73	Interferon Signaling	0.04	0.31	0.01	0.29	0.12	0.03	0.01	0.80
Smarca4	198.82	Epigenetic Regulation	0.00	0.77	0.05	0.53	0.04	0.33	0.01	0.50
Bcl2l1	154.05	Apoptosis	0.03	0.26	0.08	0.95	0.10	0.18	0.04	0.96
Ifnar1	307.14	Interferon Signaling	0.07	0.17	0.03	0.83	0.12	0.07	0.04	0.97
Nfkb1	230.49	Innate Immune Response	0.06	0.99	0.02	0.26	0.46	0.26	0.03	0.96
Eed	239.30	Epigenetic Regulation	0.29	0.02	0.04	0.98	0.08	0.02	0.13	0.79
Rpl29	1901.16	Macrophage Function	0.32	0.47	0.01	0.22	0.60	0.05	0.04	0.29
Rpl28	2352.05	Macrophage Function	0.42	0.23	0.01	0.39	0.47	0.03	0.05	0.26
Cnp	178.64	Oligodendrocyte Function	0.70	0.64	0.04	0.55	1.00	0.24	0.52	0.27
Rac1	1093.83	Growth Factor Signaling	0.15	0.69	0.01	0.13	0.83	0.28	0.04	0.69
Hdac2	245.95	Epigenetic Regulation	0.41	0.00	0.26	0.00	0.00	0.03	1.00	0.50
Pik3r2	188.01	Growth Factor Signaling	0.88	0.00	0.71	0.10	0.08	0.05	1.00	0.98
Mbd2	711.40	Epigenetic Regulation	0.45	0.01	0.56	0.07	0.06	0.23	1.00	0.75
Igf1	129.49	Macrophage-M2	0.36	0.04	0.39	0.16	0.73	0.71	0.56	1.00
Kdm3a	81.03	Epigenetic Regulation	0.31	0.02	0.54	0.43	0.09	0.13	0.64	0.99
Plxdc2	352.51	Macrophage Function	0.11	0.02	0.06	0.96	0.04	0.03	0.09	1.00
Ambra1	160.31	Autophagy	0.46	0.01	0.94	0.16	0.08	0.18	0.96	0.93
Suv39h1	50.00	Epigenetic Regulation	0.53	0.02	0.58	0.14	0.13	0.41	1.00	0.82
Gja1	263.52	Neurons and Neurotransmission	0.25	0.05	0.71	0.28	0.13	0.53	0.93	0.68
Rb1cc1	160.64	Autophagy	0.32	0.02	0.80	0.21	0.09	0.33	0.94	0.79
Dicer1	109.24	Epigenetic Regulation	0.64	0.04	0.34	0.14	0.24	0.75	0.98	0.72
Fgf13	358.12	Macrophage Function	0.01	0.21	0.38	0.99	0.38	0.42	0.26	0.04
Syp	96.21	Neurons and Neurotransmission	0.06	0.04	0.13	0.94	1.00	0.07	0.98	0.09
Hspb1	247.31	Cellular Stress	0.03	0.66	0.76	0.94	0.42	1.00	0.21	0.38
Mgmt	100.80	DNA Damage	0.04	0.62	0.76	0.93	0.53	1.00	0.26	0.46
Ltc4s	53.41	Macrophage Function	0.11	0.96	0.03	0.31	0.58	0.35	0.94	0.06
Itga7	377.02	Matrix Remodeling	0.73	0.49	0.03	0.60	0.99	0.18	0.46	0.25
Il15ra	84.56	Inflammatory Signaling	0.00	0.01	0.08	0.03	0.00	0.85	0.00	0.00
Bag4	226.77	Innate Immune Response	0.00	0.00	0.00	0.00	0.00	0.85	0.00	0.00
Kat2b	318.03	Epigenetic Regulation	0.00	0.02	0.08	0.04	0.00	0.94	0.00	0.00
Jag1	155.41	Macrophage Function	0.00	0.15	0.00	0.02	0.00	0.37	0.00	0.00
Kdm4b	78.16	Epigenetic Regulation	0.00	0.05	0.03	0.03	0.04	0.99	0.00	0.06
Ezh1	89.81	Epigenetic Regulation	0.00	0.25	0.03	0.10	0.01	0.78	0.00	0.04
Pten	799.62	Metabolism	0.00	0.07	0.08	0.08	0.00	1.00	0.00	0.00
Prkce	175.62	Growth Factor Signaling	0.00	0.09	0.01	0.03	0.02	0.81	0.00	0.07
Hsd11b1	1538.37	Metabolism	0.00	0.17	0.12	0.18	0.03	1.00	0.00	0.03
Bnip3	2411.93	Macrophage Function	0.00	0.46	0.11	0.34	0.01	0.89	0.00	0.03
Slc1a3	671.81	Neurons and Neurotransmission	0.00	0.18	0.13	0.20	0.15	1.00	0.01	0.18
Pttg1	300.46	DNA Damage	0.00	0.14	0.93	0.63	0.15	0.70	0.03	0.03
Vegfa	317.32	Macrophage Function	0.00	0.03	0.01	0.01	0.13	0.90	0.00	0.32
Jam2	235.09	Macrophage Function	0.00	0.04	0.14	0.08	0.11	0.95	0.00	0.05
PFKFB3	4284.45	Metabolism	0.00	0.63	0.04	0.24	0.01	0.55	0.00	0.05
Pink1	795.68	Autophagy	0.00	0.23	0.09	0.18	0.06	0.97	0.00	0.11
Setd1a	113.71	Epigenetic Regulation	0.00	0.18	0.14	0.20	0.25	1.00	0.01	0.29
Ncor1	408.86	Epigenetic Regulation	0.00	0.22	0.20	0.28	0.16	1.00	0.01	0.17
Gsn	22257.63	Macrophage-M2	0.00	0.23	0.17	0.27	0.16	1.00	0.01	0.19
Setd2	310.26	Epigenetic Regulation	0.00	0.04	0.80	0.30	0.45	0.46	0.03	0.06
Cyp27a1	199.28	Inflammatory Signaling	0.00	0.39	0.66	0.77	0.22	0.99	0.06	0.14
Nod1	145.52	Innate Immune Response	0.00	0.17	0.53	0.93	0.00	0.46	0.00	0.00
Tnfrsf1a	587.07	Innate Immune Response	0.00	0.32	0.36	1.00	0.01	0.51	0.01	0.00
Birc2	151.31	Innate Immune Response	0.00	0.22	0.67	0.92	0.16	0.61	0.07	0.03
Cp	622.92	Astrocyte Function	0.00	0.64	0.79	0.95	0.01	1.00	0.00	0.00
Asph	1367.22	Macrophage Function	0.00	0.52	0.47	1.00	0.00	0.75	0.00	0.00
Agt	398.27	Astrocyte Function	0.00	0.82	0.67	0.96	0.00	1.00	0.01	0.00
Bcl2	59.49	Apoptosis	0.00	0.95	0.81	1.00	0.01	1.00	0.01	0.01
Eef2k	168.35	Macrophage Function	0.00	0.61	0.88	0.96	0.03	0.99	0.02	0.02
Fkbp5	131.42	Astrocyte Function	0.00	0.94	0.53	0.97	0.04	0.95	0.07	0.02
Atg9a	145.22	Autophagy	0.00	0.25	0.54	0.97	0.05	0.57	0.03	0.01
Braf	145.86	Neurons and Neurotransmission	0.00	0.81	0.99	1.00	0.09	1.00	0.07	0.07
Uty	111.30	Epigenetic Regulation	0.00	0.37	0.21	0.41	0.01	0.99	0.10	0.01
C6	138.69	Innate Immune Response	0.00	0.19	0.73	0.87	0.01	0.60	0.02	0.03
Prkdc	72.93	Innate Immune Response	0.00	0.67	0.30	0.96	0.04	0.70	0.02	0.17
Cd163	121.89	Macrophage-M2	0.01	0.67	0.91	1.00	0.19	0.98	0.14	0.11
Steap4	1326.69	Astrocyte Function	0.01	0.76	0.64	1.00	0.10	0.94	0.08	0.21
Rgl1	84.16	Macrophage Function	0.01	0.23	0.85	0.86	0.51	0.72	0.20	0.14
Rapgef3	156.26	Autophagy	0.03	0.82	0.66	0.96	0.39	1.00	0.21	0.47
Tm4sf1	262.35	A2 Astrocyte	0.05	0.81	0.91	0.99	0.33	1.00	0.45	0.38
Shank3	66.94	Neurons and Neurotransmission	0.00	0.96	0.34	0.90	0.00	0.87	0.00	0.01
Tie1	133.06	Endothelial Cell Function	0.00	0.65	0.20	0.58	0.07	0.91	0.01	0.19
Flt1	51.28	Endothelial Cell Function	0.00	0.68	0.50	0.85	0.10	1.00	0.03	0.13
Cables1	130.17	Cell Cycle	0.01	0.51	0.85	0.98	0.30	0.92	0.19	0.13
Esam	129.80	Matrix Remodeling	0.03	0.57	0.51	0.80	0.53	1.00	0.18	0.57
Emcn	110.84	Endothelial Cell Function	0.01	0.86	0.16	0.77	0.16	0.63	0.04	0.67
Sesn2	53.79	DNA Damage	0.02	0.98	0.18	0.75	0.25	0.72	0.06	0.75
Icam2	132.00	Matrix Remodeling	0.03	0.62	0.45	0.79	0.49	1.00	0.15	0.60
Lrrc3	55.22	Macrophage Function	0.00	0.08	0.02	0.90	0.00	0.03	0.00	0.36
Bcl2l2	308.09	Apoptosis	0.01	0.84	0.12	0.71	0.15	0.54	0.03	0.73
Tspan18	60.38	Macrophage Function	0.03	0.74	0.13	0.78	0.24	0.51	0.06	0.92
Ccni	767.59	Cell Cycle	0.00	0.34	0.18	0.99	0.00	0.36	0.00	0.00
Mr1	195.53	Macrophage Function	0.00	0.66	0.97	0.99	0.00	0.98	0.00	0.00
Arhgap24	114.42	Macrophage Function	0.00	0.38	0.94	0.93	0.00	0.89	0.00	0.00
Nthl1	55.86	DNA Damage	0.00	0.63	0.25	0.63	0.00	0.95	0.02	0.00
Map1lc3a	647.91	Autophagy	0.00	0.07	0.55	0.30	0.00	0.75	0.00	0.00
AI464131	105.21	Astrocyte Function	0.00	0.45	0.84	0.89	0.00	0.98	0.00	0.00
Myct1	80.11	Endothelial Cell Function	0.00	0.15	0.42	0.37	0.00	0.95	0.00	0.00
Akt2	382.73	Growth Factor Signaling	0.00	0.66	0.75	1.00	0.00	0.94	0.00	0.00
Ifit3	250.37	Interferon Signaling	0.00	0.14	0.36	0.33	0.00	0.96	0.01	0.00
Dlg1	159.25	Neurons and Neurotransmission	0.00	0.84	0.58	0.99	0.01	0.94	0.02	0.01
Pik3r1	547.19	Metabolism	0.00	0.92	0.71	1.00	0.03	0.99	0.02	0.05
Tbc1d4	85.69	Neurons and Neurotransmission	0.00	0.79	0.61	1.00	0.03	0.94	0.02	0.06
Ldlrad3	59.65	Macrophage Function	0.00	0.17	0.93	0.68	0.12	0.75	0.02	0.03
Cox5b	2202.09	Macrophage Function	0.00	0.76	0.61	0.93	0.04	1.00	0.02	0.04
Vps4a	229.48	Neurons and Neurotransmission	0.00	0.68	0.35	0.98	0.08	0.75	0.14	0.02
Mavs	231.04	Innate Immune Response	0.00	0.57	0.68	0.89	0.14	1.00	0.05	0.12
Aldh1l1	621.30	Astrocyte Function	0.01	0.52	0.79	0.99	0.06	0.90	0.09	0.15
Ldha	3127.11	Macrophage Function	0.02	0.46	0.60	0.79	0.11	1.00	0.38	0.14
Atr	70.37	DNA Damage	0.02	0.98	0.41	0.93	0.24	0.92	0.11	0.51
Serpina3n	468.01	Astrocyte Markers	0.01	0.15	0.78	0.79	0.02	0.56	0.09	0.15
Lpl	30815.84	Metabolism	0.01	0.65	0.16	0.87	0.07	0.52	0.02	0.46
Prkaca	861.48	Growth Factor Signaling	0.02	0.96	0.30	0.88	0.22	0.84	0.08	0.59
Rnf8	76.30	DNA Damage	0.01	0.75	0.58	1.00	0.13	0.92	0.10	0.30
Clstn1	118.52	Macrophage Function	0.00	0.78	0.02	0.17	0.00	0.29	0.03	0.00
Ehmt2	556.20	Epigenetic Regulation	0.00	0.42	0.05	0.76	0.07	0.20	0.26	0.00
Ptms	1391.70	Macrophage Function	0.00	0.33	0.09	0.23	0.00	0.92	0.05	0.00
Igf1r	63.33	Growth Factor Signaling	0.00	0.11	0.04	0.06	0.01	0.96	0.32	0.00
Tarbp2	84.17	Epigenetic Regulation	0.00	0.49	0.15	0.42	0.03	0.93	0.30	0.01
Kdm4a	90.81	Epigenetic Regulation	0.00	0.28	0.17	0.30	0.01	0.99	0.19	0.01
Kdm2a	213.78	Epigenetic Regulation	0.01	0.60	0.56	0.84	0.06	1.00	0.18	0.06
Setd1b	156.84	Epigenetic Regulation	0.01	0.88	0.35	0.93	0.16	0.84	0.35	0.05
Prkar1a	775.13	Growth Factor Signaling	0.00	0.87	0.01	0.24	0.04	0.17	0.62	0.00
Casp9	99.10	Apoptosis	0.01	0.38	0.03	0.12	0.06	0.61	0.96	0.01
Rad9a	55.22	DNA Damage	0.02	0.75	0.10	0.45	0.16	0.70	0.84	0.03
Creb1	94.34	Neurons and Neurotransmission	0.03	0.94	0.10	0.55	0.30	0.61	0.95	0.05
Kat2a	100.05	Epigenetic Regulation	0.04	0.91	0.19	0.70	0.33	0.79	0.88	0.09
Suz12	242.20	Epigenetic Regulation	0.00	0.15	0.28	0.99	0.12	0.28	0.08	0.01
Kmt2c	169.30	Epigenetic Regulation	0.00	0.17	0.23	0.27	0.16	1.00	0.01	0.13
Eif1	1082.82	Epigenetic Regulation	0.00	0.65	0.12	0.80	0.14	0.45	0.44	0.02
Sirt1	98.01	Epigenetic Regulation	0.02	0.67	0.36	0.98	0.34	0.75	0.53	0.09
Kdm5b	145.72	Epigenetic Regulation	0.00	0.91	0.80	0.99	0.04	1.00	0.06	0.04
Epg5	126.65	Autophagy	0.00	0.61	0.68	1.00	0.06	0.90	0.06	0.15
Ets2	209.05	Macrophage Function	0.01	0.54	0.74	1.00	0.10	0.89	0.14	0.27
Setd7	186.22	Epigenetic Regulation	0.04	0.36	0.75	0.80	0.16	0.97	0.50	0.29
Tgm2	644.01	Macrophage-M2	0.00	0.00	0.01	0.00	0.00	0.88	0.18	0.00
Bok	63.87	Apoptosis	0.00	0.46	0.08	0.27	0.03	0.81	0.42	0.01
Dapk1	252.69	Apoptosis	0.01	0.40	0.01	0.04	0.06	0.24	1.00	0.00
Rtn4rl1	76.86	Macrophage Function	0.02	0.78	0.93	1.00	0.19	0.99	0.23	0.27
Rad17	50.08	DNA Damage	0.01	0.11	0.80	0.71	0.68	0.51	0.20	0.12
Ppp3cb	219.00	Innate Immune Response	0.00	0.05	0.86	0.50	0.68	0.38	0.12	0.08
F3	369.23	Macrophage Function	0.01	0.06	0.91	0.42	0.81	0.50	0.13	0.16
Apoe	3986.50	Macrophage-DAM Stage 1	0.02	0.05	0.92	0.44	0.98	0.37	0.28	0.23
Crebbp	286.23	Innate Immune Response	0.02	0.03	0.64	0.19	1.00	0.45	0.16	0.40
Il6ra	184.92	Macrophage Function	0.03	0.13	0.43	0.93	0.91	0.35	0.61	0.15
Slco2b1	412.32	Metabolism	0.37	0.05	0.68	0.56	0.76	0.28	0.98	0.77
Irf2	202.88	Interferon Signaling	0.27	0.05	0.87	0.35	0.85	0.46	0.78	0.89
Brd2	402.56	Epigenetic Regulation	0.01	0.02	0.35	0.08	1.00	0.50	0.06	0.41
Bnip3l	575.31	Apoptosis	0.11	0.05	0.56	0.23	0.97	0.62	0.39	0.84
Ulk1	121.30	Autophagy	0.01	0.02	0.14	0.04	0.99	0.74	0.03	0.61
Rpa1	166.53	DNA Damage	0.01	0.01	0.03	0.01	1.00	0.86	0.01	0.94
Pik3ca	214.66	Metabolism	0.08	0.00	0.03	0.01	0.54	0.81	0.04	0.96
Apc	268.60	Apoptosis	0.06	0.01	0.00	0.00	0.68	0.95	0.01	0.40
Hmgb1	390.50	Innate Immune Response	0.03	0.04	0.66	0.23	1.00	0.51	0.19	0.42
Tmcc3	205.17	Macrophage Function	0.09	0.05	0.20	0.11	0.99	0.90	0.17	0.98
Smc1a	166.37	DNA Damage	0.31	0.02	0.45	0.10	0.55	0.45	0.58	1.00
Kdm4c	77.52	Epigenetic Regulation	0.01	0.01	0.04	0.01	1.00	0.85	0.02	0.93
Pik3cb	356.11	Metabolism	0.57	0.05	0.10	0.07	0.62	0.99	0.36	0.80
Iqsec1	474.23	Autophagy	0.15	0.05	0.01	0.02	0.96	0.91	0.04	0.68
Hcar2	89.41	Macrophage Function	0.01	0.48	0.00	0.04	0.21	0.18	0.00	1.00
Pex14	155.90	Autophagy	0.01	0.20	0.02	0.07	0.64	0.75	0.01	1.00
Prkar2b	2986.68	Growth Factor Signaling	0.29	0.67	0.04	0.26	0.96	0.54	0.13	0.81
Nbn	69.57	DNA Damage	0.03	0.12	0.44	0.34	0.91	0.91	0.14	0.58
Cflar	347.94	Innate Immune Response	0.32	0.05	0.23	0.12	0.79	0.87	0.40	1.00
Jarid2	78.29	Epigenetic Regulation	0.07	0.10	0.01	0.03	1.00	0.80	0.02	0.88
Bid	87.90	Innate Immune Response	0.01	0.04	0.13	0.96	0.90	0.08	0.66	0.03
Abl1	107.99	Growth Factor Signaling	0.01	0.05	0.24	0.88	0.92	0.13	0.55	0.05
Casp7	105.88	Innate Immune Response	0.03	0.39	0.22	0.99	0.60	0.44	0.76	0.08
H2-T23	91.42	Innate Immune Response	0.99	0.33	0.01	0.06	0.88	0.45	0.18	0.18
Rpl9	2302.61	Macrophage Function	0.00	0.78	0.00	0.01	0.05	0.02	0.82	0.00
Ep300	228.89	Innate Immune Response	0.01	0.61	0.00	0.16	0.22	0.05	0.99	0.00
Rps10	562.48	Macrophage Function	0.35	0.85	0.02	0.25	0.94	0.17	0.50	0.08
Gadd45a	180.23	DNA Damage	0.16	0.34	0.00	0.20	0.98	0.03	0.33	0.02
Mertk	167.12	Macrophage-Homeostatic	0.04	0.65	0.01	0.24	0.55	0.09	0.88	0.01
Fgl2	100.72	Inflammatory Signaling	0.57	0.82	0.02	0.19	0.93	0.29	0.40	0.13
Dusp7	156.52	Macrophage Function	0.85	0.73	0.00	0.06	0.98	0.03	0.04	0.05
Nlgn2	71.82	Neurons and Neurotransmission	0.85	0.71	0.01	0.10	1.00	0.20	0.12	0.17
H2afx	116.01	Epigenetic Regulation	0.23	0.02	0.30	0.66	0.74	0.10	1.00	0.38
Ifitm2	1243.52	Interferon Signaling	0.44	0.41	0.00	0.01	0.65	0.07	0.06	0.01
Cd24a	55.23	Adaptive Immune Response	0.12	0.05	0.01	0.02	0.08	0.84	0.64	0.03

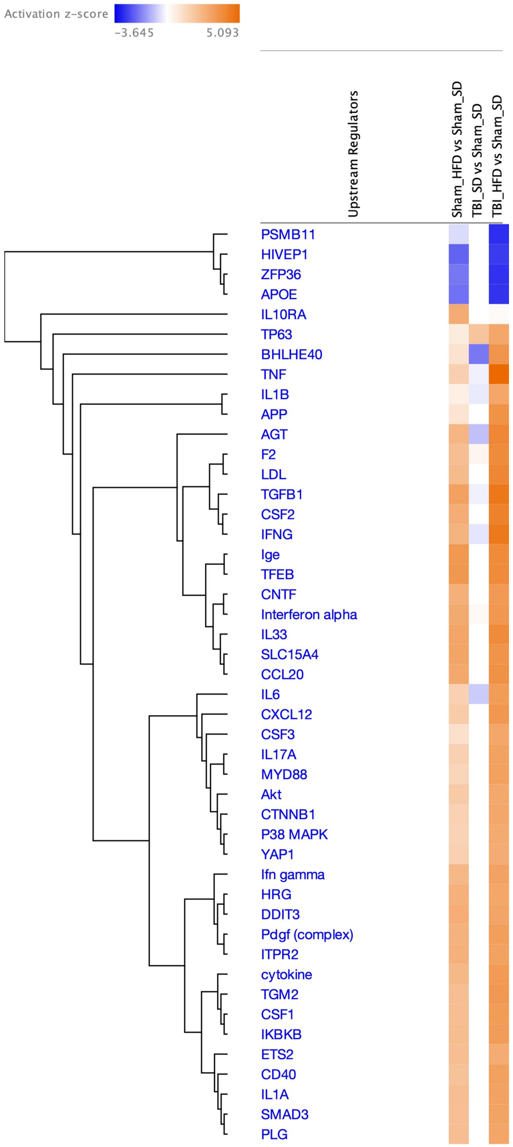

Upstream regulators – Genes (upregulated)

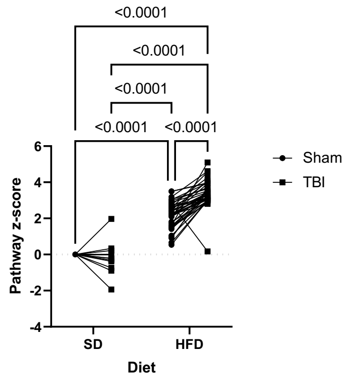

Source of Variation	% of total variation	P value	P value summary	Significant?	
Diet	79.66	<0.0001	****	Yes	
Injury	3.446	<0.0001	****	Yes	
Diet x Injury	4.076	<0.0001	****	Yes	
Pathway x Diet	3.955				
Pathway x Injury	2.456				
Pathway	3.364				
ANOVA table	SS	DF	MS	F (DFn, DFd)	P value
Diet	331.5	1	331.5	F (1, 41) = 825.9	P<0.0001
Injury	14.34	1	14.34	F (1, 41) = 57.52	P<0.0001
Diet x Injury	16.96	1	16.96	F (1, 41) = 54.95	P<0.0001

Upstream regulators – Genes (downregulated)

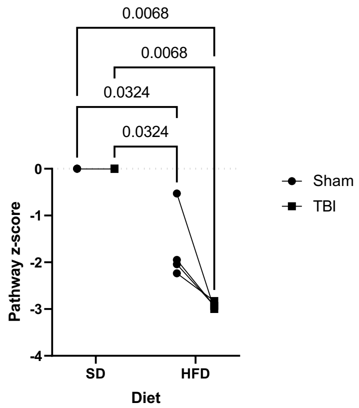

Source of Variation	% of total variation	P value	P value summary	Significant?	
Diet	81.28	0.0011	**	Yes	
Injury	5.814	0.0611	ns	No	
Diet x Injury	5.814	0.0611	ns	No	
Pathway x Diet	1.511				
Pathway x Injury	2.035				
Pathway	1.511				
ANOVA table	SS	DF	MS	F (DFn, DFd)	P value
Diet	21.26	1	21.26	F (1, 3) = 161.4	P=0.0011
Injury	1.521	1	1.521	F (1, 3) = 8.572	P=0.0611
Diet x Injury	1.521	1	1.521	F (1, 3) = 8.572	P=0.0611

Figure 3-Supplemental

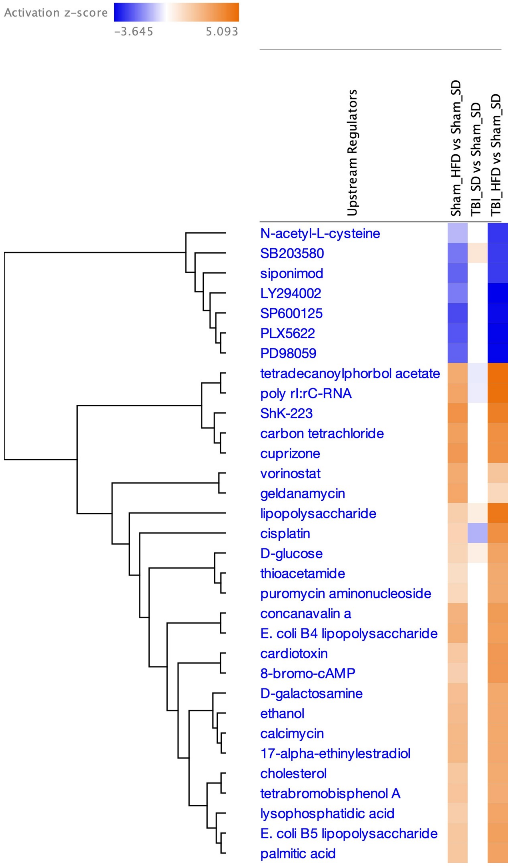

Upstream regulators – Drugs (upregulated)

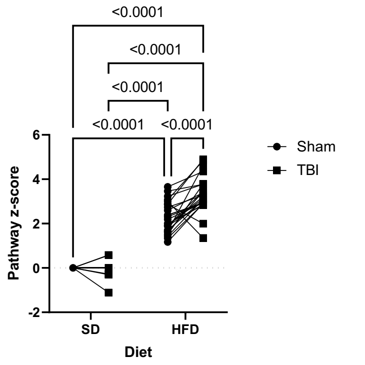

Source of Variation	% of total variation	P value	P value summary	Significant?	
Diet	82.22	<0.0001	****	Yes	
Injury	2.685	<0.0001	****	Yes	
Diet x Injury	2.918	<0.0001	****	Yes	
Pathway x Diet	3.833				
Pathway x Injury	2.423				
Pathway	3.328				
ANOVA table	SS	DF	MS	F (DFn, DFd)	P value
Diet	196.3	1	196.3	F (1, 24) = 514.8	P<0.0001
Injury	6.408	1	6.408	F (1, 24) = 26.59	P<0.0001
Diet x Injury	6.965	1	6.965	F (1, 24) = 27.00	P<0.0001

Upstream regulators – Drugs (downregulated)

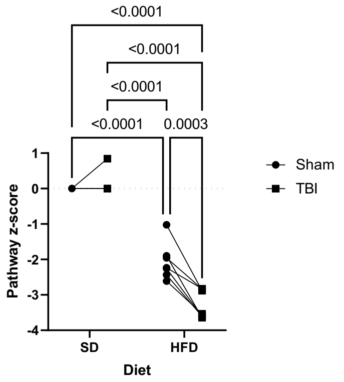

Source of Variation	% of total variation	P value	P value summary	Significant?	
Diet	86.08	<0.0001	****	Yes	
Injury	3.468	0.0041	**	Yes	
Diet x Injury	5.159	0.0003	***	Yes	
Pathway x Diet	1.478				
Pathway x Injury	1.028				
Pathway	2.240				
ANOVA table	SS	DF	MS	F (DFn, DFd)	P value
Diet	52.09	1	52.09	F (1, 6) = 349.5	P<0.0001
Injury	2.099	1	2.099	F (1, 6) = 20.23	P=0.0041
Diet x Injury	3.122	1	3.122	F (1, 6) = 56.21	P=0.0003

Figure 4-Supplemental

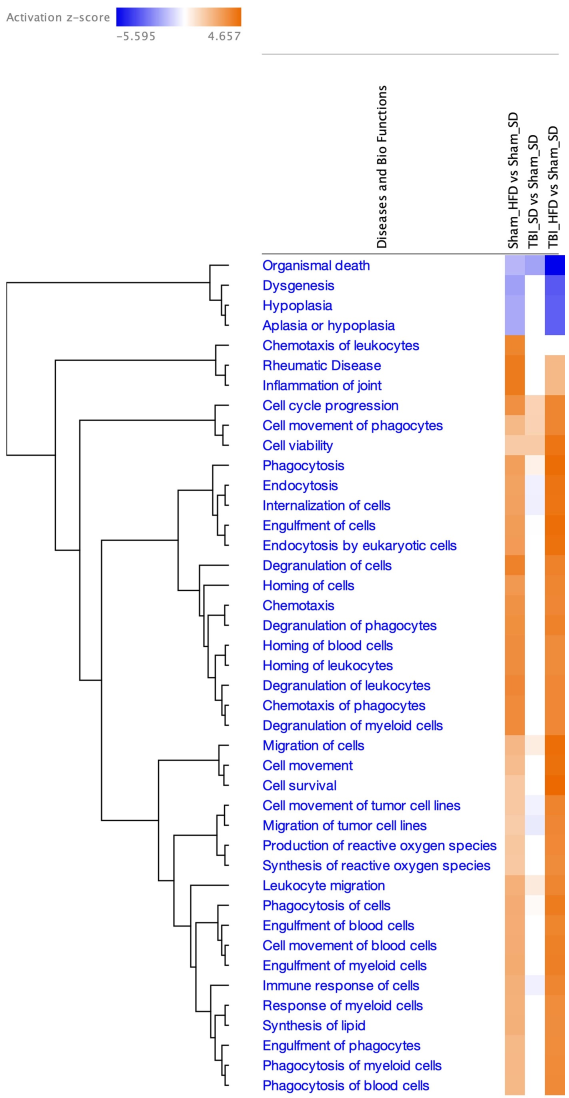

Diseases (upregulated)

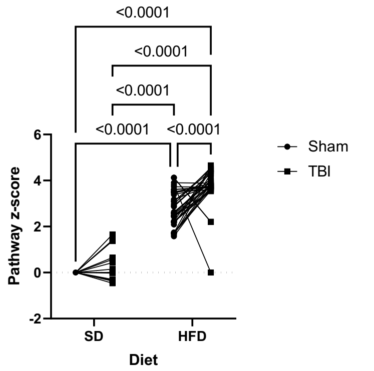

Source of Variation	% of total variation	P value	P value summary	Significant?	
Diet	84.80	<0.0001	****	Yes	
Injury	2.318	<0.0001	****	Yes	
Diet x Injury	1.414	0.0005	***	Yes	
Pathway x Diet	1.821				
Pathway x Injury	4.205				
Pathway	1.856				
ANOVA table	SS	DF	MS	F (DFn, DFd)	P value
Diet	388.5	1	388.5	F (1, 37) = 1723	P<0.0001
Injury	10.62	1	10.62	F (1, 37) = 20.39	P<0.0001
Diet x Injury	6.479	1	6.479	F (1, 37) = 14.59	P=0.0005

Diseases (downregulated)

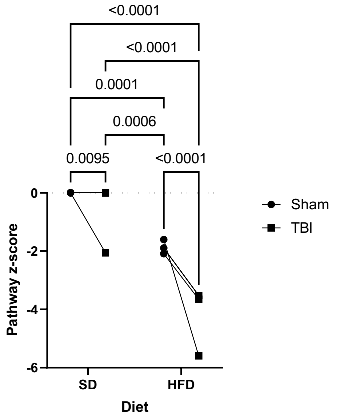

Source of Variation	% of total variation	P value	P value summary	Significant?	
Diet	63.91	<0.0001	****	Yes	
Injury	16.03	0.0920	ns	No	
Diet x Injury	6.186	0.0003	***	Yes	
Pathway x Diet	0.09986				
Pathway x Injury	8.034				
Pathway	5.696				
ANOVA table	SS	DF	MS	F (DFn, DFd)	P value
Diet	29.47	1	29.47	F (1, 3) = 1920	P<0.0001
Injury	7.390	1	7.390	F (1, 3) = 5.984	P=0.0920
Diet x Injury	2.853	1	2.853	F (1, 3) = 413.2	P=0.0003

Figure 5-Supplemental

Figure 6A-Supplemental

HIPPOCAMPUS

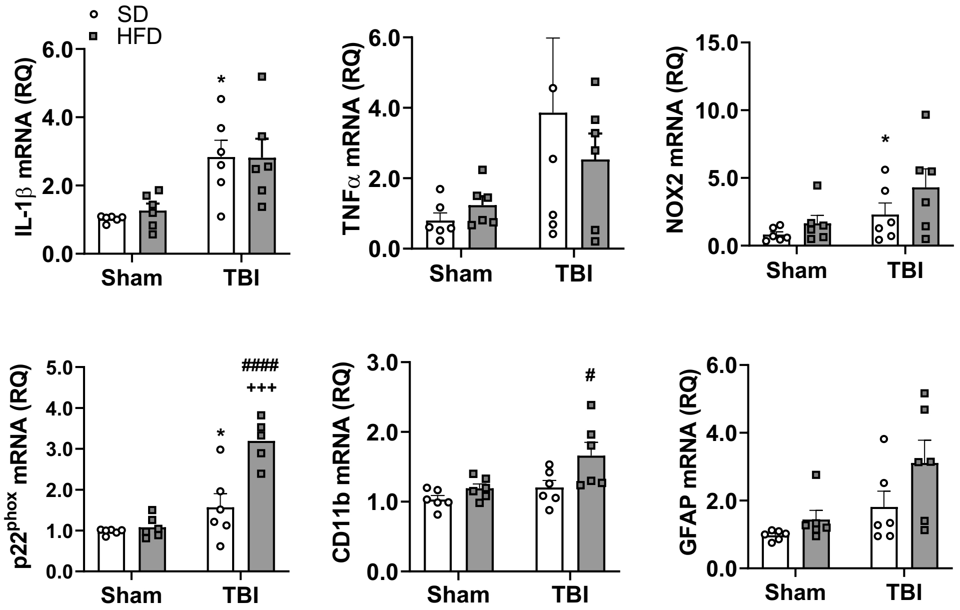

Figure 6B-Supplemental

FRONTAL CORTEX

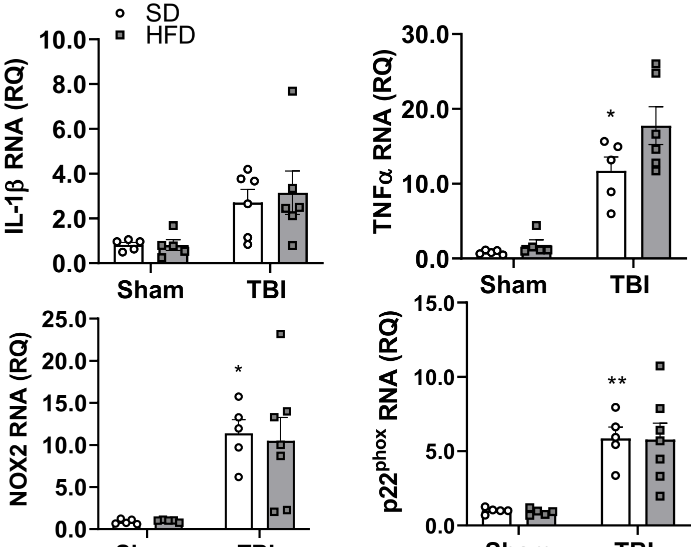

Figure 7-Supplemental

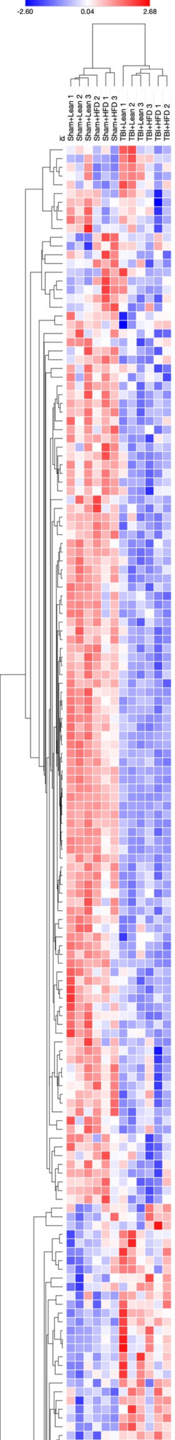

Symbol	Average	Probe.Annotation	p_val_Diet	p_val_Condition	p_val_diet:condition	p_TBILean:ShamLean	p_ShamHFD:ShamLean	p_TBIHFD:ShamHFD	p_TBIHFD:ShamLean	p_TBIHFD:TBILean
Cd34	2237.20	Cell Migration	0.03	0.52	0.23	0.54	0.81	0.97	0.57	0.10
Pccb	333.62	Lipid Metabolism	0.02	0.01	0.51	0.10	0.50	0.38	0.99	0.15
Pros1	1341.40	Complement System	0.00	0.03	0.87	0.35	0.03	0.26	0.45	0.04
Nptn	2270.57	Neurogenesis	0.00	0.09	0.32	0.23	0.06	0.93	0.15	0.01
Cdc42	7587.42	Neurogenesis	0.04	0.06	0.87	0.39	0.42	0.51	1.00	0.32
Bub3	420.11	Cell Cycle	0.01	0.61	0.21	0.94	0.57	0.57	0.11	0.05
Sgce	1063.86	Cytoskeletal Dynamics	0.04	0.08	0.57	0.74	0.59	0.31	0.05	0.22
Hexb	77311.57	Microglia-Homeostatic	0.02	0.37	0.53	0.68	0.13	1.00	0.09	0.42
Lgmn	35051.20	Microglia Markers	0.00	0.05	0.38	0.17	0.01	0.75	0.00	0.05
Psmc2	790.77	Wnt Signaling	0.05	0.12	0.48	0.36	0.21	0.89	0.08	0.69
Pias1	221.04	JAK-STAT	0.01	0.34	0.02	0.50	0.01	0.08	0.35	0.99
Adamts1	1734.37	Microglia Markers	0.06	0.48	0.03	0.15	0.04	0.54	0.24	0.98
Reck	51.11	Blood Brain Barrier	0.64	0.04	0.18	0.89	0.55	0.09	0.53	0.90
Slc25a12	173.35	Myelogenesis	0.74	0.01	0.01	1.00	0.07	0.00	0.18	0.15
Tie1	61.47	Endothelial Cell Function	0.69	0.88	0.02	0.18	0.31	0.25	1.00	0.14
Mkrn1	837.28	Antigen Processing and Presentation	0.03	0.16	0.01	0.55	0.01	0.03	0.87	0.93
Icam2	69.15	Cell Migration	0.57	0.23	0.04	0.85	0.22	0.11	0.96	0.58
Pak1	500.62	T-cell signaling	0.23	0.02	0.16	0.77	0.26	0.05	0.68	1.00
Stmn1	198.90	Neuronal Markers	0.03	0.10	0.42	0.89	0.13	0.29	0.93	0.58
Mtmr4	135.51	Autophagy	0.20	0.02	0.32	0.10	0.99	0.64	0.77	0.37
Ticam1	167.68	NO Metabolism and Signaling	0.20	0.07	0.04	0.05	0.85	0.99	0.96	0.09
Fos	10885.67	MAPK & PI3K	0.30	0.04	0.45	0.65	1.00	0.17	0.13	0.56
Camk2d	711.71	Calcium Signaling	0.35	0.02	0.02	0.02	0.11	1.00	0.10	0.61
Clcf1	232.49	A2 Astrocyte	0.91	0.02	0.34	0.55	0.92	0.09	0.21	0.85
Nlrp1b	117.14	Inflammasome	0.03	0.00	0.99	0.09	0.32	0.09	0.81	0.31
Klrd1	92.56	Innate Immune Response	0.19	0.03	0.42	0.63	0.97	0.15	0.08	0.42
Trim2	165.59	Interferon Signaling	0.94	0.05	0.94	0.42	1.00	0.37	0.37	1.00
Fcgrt	612.78	Astrocyte Markers	0.90	0.00	0.86	0.08	1.00	0.05	0.07	1.00
Orc4	357.77	Cell Cycle	0.66	0.00	0.96	0.08	0.99	0.08	0.05	0.98
Glul	11142.06	Glutamatergic Synapse	0.39	0.00	0.67	0.00	0.99	0.00	0.00	0.78
Trim26	369.14	Interferon Signaling	0.71	0.00	0.38	0.00	0.98	0.03	0.02	0.79
Stab1	748.57	Microglia-M2	0.88	0.00	0.13	0.01	0.71	0.35	0.08	0.59
Kcnd1	129.19	Ion Transport	0.86	0.00	0.88	0.07	1.00	0.10	0.10	1.00
Pik3r1	1353.58	MAPK & PI3K	0.71	0.00	0.77	0.05	1.00	0.09	0.10	0.96
Atp2b2	63.29	Ion Transport	0.42	0.01	0.39	0.39	0.61	0.07	0.38	1.00
Pecam1	273.61	Blood Brain Barrier	0.52	0.00	0.05	0.19	0.24	0.00	0.04	0.68
Camk2g	146.85	Calcium Signaling	0.46	0.00	0.65	0.12	1.00	0.05	0.04	0.82
Ccr5	3460.55	Primed Microglia	0.70	0.00	0.48	0.12	0.99	0.03	0.04	0.85
Stxbp1	103.95	Neurogenesis	0.74	0.00	0.51	0.03	0.88	0.01	0.02	0.99
Scoc	3003.06	Primed Microglia	1.00	0.03	0.63	0.44	0.98	0.18	0.28	0.98
Tnfrsf17	168.96	Cytokines	0.78	0.01	0.99	0.15	1.00	0.15	0.20	1.00
Il18	291.30	Cytokines	0.78	0.01	0.59	0.18	1.00	0.06	0.08	0.93
Basp1	1180.28	Cytoskeletal Dynamics	0.68	0.00	0.58	0.07	1.00	0.02	0.02	0.89
Nuak1	359.40	Primed Microglia	0.73	0.00	0.28	0.02	0.71	0.00	0.01	0.94
Bin1	4118.82	Microglia-Homeostatic	0.07	0.00	0.37	0.01	0.22	0.00	0.02	0.86
Tanc2	1622.34	Cytoskeletal Dynamics	0.75	0.00	0.39	0.01	0.81	0.06	0.02	0.97
Tjp1	1213.62	Gap Junctions	0.57	0.00	0.45	0.00	1.00	0.00	0.00	0.76
Agmo	736.25	Lipid Metabolism	0.37	0.00	0.61	0.02	0.73	0.04	0.01	0.99
Ggta1	464.13	A1 Astrocyte	0.59	0.00	0.26	0.00	0.61	0.02	0.00	0.96
Prkce	133.93	Phagocytosis	0.59	0.00	0.34	0.00	0.69	0.01	0.00	0.99
Mef2a	2304.30	Microglia-Homeostatic	0.05	0.00	0.23	0.01	0.13	0.09	0.00	0.89
Cdk7	529.88	Cell Cycle	0.03	0.00	0.78	0.03	0.37	0.02	0.00	0.22
Cables1	955.94	Cell Cycle	0.11	0.00	0.54	0.01	0.38	0.02	0.00	0.85
Egr1	15802.23	Microglia-Homeostatic	0.41	0.00	0.23	0.02	0.47	0.22	0.03	0.99
Ckb	4250.45	Astrocyte Markers	0.75	0.00	0.52	0.01	0.99	0.02	0.02	0.89
Tmem100	80.34	Astrocyte Markers	0.21	0.00	0.53	0.07	0.96	0.02	0.01	0.52
Jun	20389.28	Microglia Markers	0.32	0.00	0.90	0.01	0.91	0.01	0.00	0.83
Crot	401.40	Lipid Metabolism	0.35	0.00	0.96	0.05	0.88	0.05	0.02	0.90
Cacnb2	116.27	MAPK & PI3K	0.14	0.00	0.34	0.02	0.97	0.00	0.00	0.31
Gpr34	9652.79	Microglia-Homeostatic	0.16	0.00	0.71	0.02	0.85	0.01	0.00	0.56
Siglech	7087.94	Microglia-Homeostatic	0.43	0.00	0.34	0.00	0.58	0.01	0.00	1.00
E2f3	250.78	Cell Cycle	0.27	0.00	0.51	0.00	0.98	0.00	0.00	0.58
Ctsf	1594.79	Microglia Markers	0.00	0.00	0.01	0.00	0.00	0.00	0.00	0.27
Slco2b1	1071.17	Microglia Markers	0.01	0.00	0.02	0.00	0.01	0.00	0.00	1.00
Csf1r	23290.91	Microglia-Homeostatic	0.02	0.00	0.05	0.00	0.03	0.00	0.00	0.97
Sall1	3347.39	Microglia-Homeostatic	0.01	0.00	0.06	0.00	0.01	0.00	0.00	0.66
Tgfbr1	4126.54	Microglia-Homeostatic	0.07	0.00	0.49	0.00	0.27	0.00	0.00	0.77
Slc7a8	1048.15	Ion Transport	0.00	0.00	0.00	0.00	0.00	0.00	0.00	0.99
Elmo1	1303.74	Primed Microglia	0.02	0.00	0.02	0.00	0.02	0.00	0.00	1.00
Cx3cr1	14097.44	Microglia-Homeostatic	0.00	0.00	0.13	0.00	0.02	0.00	0.00	0.45
Itgam	5262.64	Microglia-Homeostatic	0.15	0.00	0.41	0.00	0.36	0.01	0.00	0.95
Cttnbp2nl	659.52	Microglia Markers	0.03	0.00	0.05	0.00	0.03	0.00	0.00	1.00
Pmepa1	1020.40	Microglia-Homeostatic	0.06	0.00	0.07	0.00	0.06	0.00	0.00	1.00
P2ry12	19247.80	Microglia-Homeostatic	0.04	0.00	0.29	0.00	0.14	0.00	0.00	0.81
Slc2a5	4454.63	Microglia-Homeostatic	0.09	0.00	0.30	0.00	0.22	0.00	0.00	0.93
Smad3	227.80	Microglia-Homeostatic	0.60	0.00	0.40	0.00	0.75	0.00	0.00	0.99
Rtn1	324.68	Neurogenesis	0.98	0.00	0.21	0.00	0.76	0.00	0.00	0.78
Sparc	11515.38	Microglia-Homeostatic	0.07	0.00	0.31	0.00	0.18	0.00	0.00	0.88
P2ry13	3855.25	Microglia-Homeostatic	0.29	0.00	0.76	0.00	0.73	0.00	0.00	0.94
Prkab1	811.95	Glucose Metabolism	0.25	0.00	0.07	0.00	0.16	0.00	0.00	0.93
Stambpl1	506.90	Primed Microglia	0.17	0.00	0.37	0.00	0.36	0.01	0.00	0.98
Adgrg1	554.46	Microglia-Homeostatic	0.99	0.00	0.60	0.00	0.98	0.00	0.00	0.98
Tmem119	1299.18	Microglia-Homeostatic	0.70	0.00	0.99	0.01	0.99	0.01	0.00	0.99
Entpd1	2851.46	Microglia-Homeostatic	1.00	0.00	0.69	0.01	0.99	0.02	0.01	0.99
Cep68	267.01	Primed Microglia	0.97	0.00	0.16	0.00	0.68	0.03	0.01	0.71
Etv5	653.38	Neurogenesis	0.24	0.00	0.16	0.01	0.27	0.10	0.01	1.00
Tlr3	270.68	NF-kappaB Signaling	0.81	0.00	0.34	0.03	0.81	0.22	0.06	0.95
Serinc3	15325.12	Primed Microglia	0.14	0.00	0.16	0.00	0.19	0.06	0.00	1.00
Mertk	2059.26	Microglia-Homeostatic	0.21	0.00	0.08	0.00	0.16	0.02	0.00	0.97
Crkl	219.40	Neurotrophin Signaling	0.04	0.00	0.07	0.01	0.05	0.52	0.01	0.99
Mafb	2263.07	Microglia-Homeostatic	0.95	0.01	0.23	0.03	0.78	0.38	0.11	0.82
Usp2	1286.63	Microglia Markers	0.03	0.00	0.28	0.00	0.10	0.03	0.00	0.70
Cdk5r1	124.53	Neurotrophin Signaling	0.14	0.00	0.64	0.06	0.84	0.02	0.01	0.47
Ppm1l	116.10	Primed Microglia	0.12	0.00	0.69	0.02	0.47	0.04	0.01	0.79
Plxdc2	4537.41	Microglia Markers	0.02	0.01	0.22	0.04	0.07	0.47	0.01	0.69
Map2k1	1033.94	MAPK & PI3K	0.31	0.00	0.38	0.02	0.52	0.10	0.01	1.00
Mapk14	1061.74	MAPK & PI3K	0.15	0.00	0.57	0.01	0.45	0.05	0.01	0.89
Nos1ap	147.24	Oxidative & Nitrosative Stress	0.31	0.00	0.09	0.01	0.23	0.19	0.01	0.94
Il10ra	3397.40	Cytokines	0.02	0.00	0.12	0.01	0.04	0.14	0.00	0.83
F11r	3080.16	Primed Microglia	0.00	0.00	0.46	0.00	0.02	0.01	0.00	0.10
Abcc3	604.17	Oxidative & Nitrosative Stress	0.04	0.00	0.03	0.00	0.03	0.15	0.00	1.00
Sirpa	5845.38	NO Metabolism and Signaling	0.00	0.00	0.08	0.01	0.01	0.32	0.00	0.32
Slc46a1	296.74	Ion Transport	0.38	0.02	0.33	0.08	0.53	0.52	0.09	1.00
Olfml3	6107.95	Microglia-Homeostatic	0.25	0.00	0.60	0.15	0.96	0.05	0.03	0.60
Fcrls	11065.30	Microglia-Homeostatic	0.46	0.01	0.99	0.10	0.94	0.10	0.05	0.95
Calm2	1559.96	Calcium Signaling	0.62	0.01	0.71	0.20	0.92	0.09	0.23	1.00
Cst3	183431.23	Microglia-Homeostatic	0.78	0.01	0.66	0.25	0.95	0.11	0.22	1.00
Gng2	888.44	Serotonergic Synapse	0.41	0.01	0.41	0.35	0.63	0.07	0.35	1.00
Ltc4s	240.93	Microglia Markers	0.64	0.03	0.94	0.28	0.99	0.32	0.45	0.98
Rhob	2475.08	Primed Microglia	0.71	0.00	1.00	0.07	0.99	0.07	0.10	0.99
Adrb1	225.98	Neuroactive Ligands and Receptors	0.26	0.00	0.89	0.07	0.77	0.06	0.21	0.87
Ap3m2	159.39	Primed Microglia	0.76	0.02	0.38	0.10	0.82	0.52	0.18	0.97
Dnajb9	869.26	Proteotoxic Stress	0.96	0.02	0.25	0.07	0.84	0.66	0.26	0.80
Hacd3	275.15	Lipid Metabolism	0.04	0.02	0.98	0.27	0.40	0.26	0.03	0.38
Dagla	190.54	Neurogenesis	0.08	0.00	0.22	0.15	0.96	0.01	0.01	0.16
B4galt6	443.22	Oligodendrocyte Differentiation	0.71	0.04	0.05	1.00	0.55	0.04	0.28	0.30
Adora3	634.29	Microglia-Homeostatic	0.32	0.03	0.95	0.28	0.86	0.32	0.11	0.89
Rab6b	479.49	Primed Microglia	0.62	0.01	0.48	0.06	0.82	0.24	0.07	1.00
Ywhah	2798.78	Neurogenesis	0.77	0.04	0.74	0.46	0.97	0.25	0.44	1.00
Atp8a2	816.29	Primed Microglia	0.34	0.00	0.34	0.21	1.00	0.03	0.03	0.52
Lpar6	889.95	Purinergic Signaling	0.74	0.03	0.67	0.42	1.00	0.19	0.21	0.94
Gphn	80.37	GABAergic Synapse	0.03	0.42	0.37	1.00	0.65	0.61	0.14	0.13
Pdha1	263.32	Glucose Metabolism	0.03	0.12	0.97	0.65	0.30	0.62	0.05	0.28
Rnasel	282.95	Interferon Signaling	0.01	0.18	0.20	1.00	0.40	0.25	0.02	0.03
Psmd10	150.67	Antigen Processing and Presentation	0.50	0.02	0.01	0.01	0.33	0.99	0.47	0.09
Nes	85.66	Cytoskeletal Dynamics	0.77	0.05	0.05	0.04	0.51	1.00	0.52	0.31
Chchd3	203.02	Glucose Metabolism	0.85	0.02	0.09	0.04	0.64	0.91	0.31	0.48
Ap2m1	1436.19	Gap Junctions	0.51	0.02	0.03	0.02	0.18	0.99	0.12	0.58
Lta4h	355.30	Lipid Metabolism	0.93	0.03	0.01	0.01	0.17	0.97	0.31	0.15
Calm1	1579.55	Calcium Signaling	0.31	0.02	0.57	0.47	0.98	0.16	0.10	0.65
Kctd12	815.88	GABAergic Synapse	0.05	0.05	0.22	0.13	0.12	0.90	0.05	0.89
S100b	56.40	Astrocyte Markers	0.47	0.02	0.35	0.59	0.63	0.10	0.50	1.00
Ahcyl1	424.70	Astrocyte Markers	0.86	0.03	0.77	0.23	1.00	0.40	0.36	0.98
Lamtor3	487.58	Autophagy	0.20	0.00	0.06	0.00	0.94	0.16	0.07	0.13
Akt3	255.87	MAPK & PI3K	0.45	0.00	0.35	0.01	1.00	0.04	0.04	0.61
Mog	73.55	Oligodendrocyte Markers	0.67	0.01	0.39	0.08	0.78	0.43	0.12	0.99
Plp1	974.39	Oligodendrocyte Markers	0.55	0.04	0.63	0.22	0.86	0.52	0.20	1.00
Ermn	63.14	Oligodendrocyte Markers	0.69	0.04	0.56	0.21	0.89	0.57	0.24	1.00
Iigp1	78.56	Interferon Signaling	0.47	0.01	0.45	0.09	1.00	0.41	0.39	0.70
Mapt	50.13	Neurogenesis	0.52	0.04	0.19	0.09	0.94	0.88	0.59	0.48
Inpp5f	156.89	Neurogenesis	0.92	0.04	0.04	0.04	0.36	1.00	0.33	0.42
Kpna1	313.34	Interferon Signaling	0.36	0.05	0.41	0.18	1.00	0.72	0.76	0.59
Ptpn1	1448.95	Interferon Signaling	0.30	0.03	0.69	0.43	0.71	0.20	0.69	0.96
Tnfrsf11a	612.49	Cytokines	0.67	0.04	0.95	0.32	0.99	0.37	0.49	0.98
Hadh	198.57	Lipid Metabolism	0.47	0.01	0.75	0.12	0.99	0.23	0.34	0.87
Fgf13	96.85	Neurogenesis	0.30	0.00	0.62	0.01	0.97	0.03	0.05	0.67
Opa1	246.13	Neurogenesis	0.21	0.01	0.17	0.02	1.00	0.45	0.40	0.25
Atp5g3	408.73	Glucose Metabolism	0.38	0.01	0.79	0.09	0.83	0.15	0.05	0.97

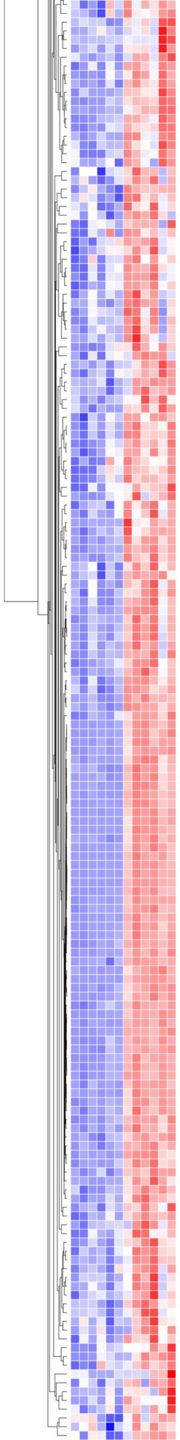

Rapgef2	82.10	MAPK & PI3K	0.52	0.01	0.66	0.12	0.85	0.30	0.10	1.00
Ptpn11	376.02	Neurotrophin Signaling	0.60	0.01	0.77	0.22	1.00	0.12	0.09	0.93
Rab1a	2571.69	Autophagy	0.63	0.01	0.15	0.57	0.86	0.03	0.09	0.49
Gls	1318.50	Glutamatergic Synapse	0.15	0.05	0.41	0.74	0.96	0.18	0.09	0.36
Igfbp5	52.60	Insulin Signaling	0.07	0.02	0.11	0.87	1.00	0.04	0.03	0.10
Pkd2	148.52	NO Metabolism and Signaling	0.47	0.01	0.35	0.38	1.00	0.06	0.07	0.62
Stat3	940.99	Interferon Signaling	0.66	0.01	0.47	0.26	1.00	0.06	0.08	0.82
Nsf	449.58	GABAergic Synapse	1.00	0.00	0.80	0.03	1.00	0.05	0.04	1.00
Diaph1	345.90	Cell Migration	0.60	0.00	0.46	0.01	0.79	0.06	0.02	1.00
Abca1	3323.12	Lipid Metabolism	0.80	0.00	0.86	0.01	1.00	0.01	0.00	0.99
Jak2	280.11	JAK-STAT	0.27	0.00	0.26	0.04	1.00	0.00	0.00	0.39
Vegfa	222.71	Blood Brain Barrier	0.04	0.00	0.69	0.00	0.49	0.00	0.00	0.24
B4galt5	142.66	Oligodendrocyte Differentiation	0.03	0.00	0.11	0.01	0.95	0.00	0.00	0.06
Olfr110	205.80	Microglia Markers	0.21	0.00	0.52	0.01	0.52	0.02	0.00	0.96
Gsn	186.35	Oligodendrocyte Function	0.03	0.00	0.95	0.00	0.31	0.00	0.00	0.34
Pik3cb	705.03	MAPK & PI3K	0.74	0.00	0.78	0.04	0.97	0.06	0.03	1.00
Ranbp9	598.95	Neurogenesis	0.36	0.01	0.86	0.13	0.94	0.09	0.04	0.85
Gnb1	1038.44	Serotonergic Synapse	0.69	0.01	0.92	0.13	1.00	0.11	0.08	0.98
Cltc	6791.66	Wnt Signaling	0.98	0.03	0.95	0.36	1.00	0.32	0.35	1.00
Psma5	446.22	Antigen Processing and Presentation	0.23	0.03	0.84	0.26	0.87	0.37	0.78	0.72
Arl6ip5	828.66	Glutamatergic Synapse	0.40	0.01	0.76	0.22	0.83	0.12	0.37	0.98
Atg5	506.13	Autophagy	0.13	0.00	0.36	0.02	0.31	0.00	0.04	0.96
Serpine2	3622.13	Microglia-DAM Stage 2-MGnD	0.02	0.00	0.43	0.08	0.11	0.01	0.50	0.50
Prkar1a	1647.89	Insulin Signaling	0.23	0.01	0.30	0.31	0.38	0.04	0.36	1.00
Adar	228.97	Interferon Signaling	0.15	0.01	0.88	0.15	0.63	0.11	0.52	0.75
Eloc	1026.23	Antigen Processing and Presentation	0.58	0.02	0.39	0.10	0.73	0.52	0.14	0.99
Slc25a4	1003.41	Calcium Signaling	0.69	0.03	0.20	0.09	0.60	0.85	0.23	0.90
Ndufb5	259.56	Cannabinoid Signaling	0.24	0.00	0.02	0.00	0.07	0.64	0.01	0.67
Sugt1	633.40	Inflammasome	0.22	0.01	0.10	0.03	0.20	0.77	0.05	0.98
Acot13	327.90	Lipid Metabolism	0.88	0.01	0.67	0.12	1.00	0.28	0.22	0.97
Atp5pb	702.68	Glucose Metabolism	0.82	0.00	0.22	0.02	0.70	0.24	0.05	0.87
Atp6v1e1	610.66	Phagocytosis	0.72	0.01	0.23	0.03	0.91	0.41	0.18	0.66
Sod1	1529.52	Oxidative & Nitrosative Stress	0.32	0.00	0.07	0.00	0.19	0.11	0.01	0.87
Ndufa10	422.29	Cannabinoid Signaling	0.25	0.01	0.17	0.04	1.00	0.61	0.51	0.29
Ctss	54564.60	Microglia-Homeostatic	0.05	0.00	0.27	0.01	0.86	0.14	0.39	0.14
Aif1	6414.13	Microglia-M1	0.58	0.04	0.29	0.14	0.98	0.80	0.59	0.64
Tmsb4x	94436.75	Microglia-Homeostatic	0.66	0.01	0.28	0.05	0.96	0.45	0.25	0.67
Clta	4982.53	Wnt Signaling	0.49	0.01	0.11	0.02	0.87	0.64	0.27	0.35
Abcc8	52.36	Neuronal Markers	0.04	0.00	0.03	0.00	1.00	0.54	0.47	0.03
Cpne2	81.83	Astrocyte Markers	0.57	0.00	0.12	0.01	0.40	0.28	0.03	0.84
Atp5b	1137.73	Glucose Metabolism	0.74	0.02	0.63	0.15	0.93	0.39	0.18	1.00
Nlrp3	1241.49	Inflammasome	0.11	0.00	0.19	0.09	0.99	0.01	0.00	0.18
Panx1	73.20	Inflammasome	0.08	0.00	0.78	0.05	0.63	0.03	0.01	0.41
Sqstm1	2394.81	Autophagy	0.57	0.00	0.24	0.11	0.96	0.01	0.02	0.58
C3ar1	3811.69	Primed Microglia	0.69	0.00	0.53	0.06	0.87	0.02	0.05	1.00
Gas6	1285.50	Neurogenesis	0.92	0.00	0.82	0.11	1.00	0.07	0.08	0.99
C9orf72	466.04	Primed Microglia	0.65	0.00	0.61	0.02	1.00	0.01	0.01	0.89
Calm3	551.91	Calcium Signaling	0.16	0.00	0.50	0.02	0.44	0.06	0.01	0.93
Cd244a	59.86	Adaptive Immune Response	0.95	0.00	0.33	0.01	0.86	0.05	0.02	0.90
Eid1	540.40	Primed Microglia	0.90	0.00	0.32	0.00	0.91	0.01	0.01	0.83
Gabarapl1	598.34	GABAergic Synapse	0.11	0.00	0.15	0.00	0.15	0.02	0.00	1.00
Gnai1	67.21	Serotonergic Synapse	0.06	0.00	0.16	0.00	0.11	0.04	0.00	0.97
Flt1	155.42	Endothelial Cell Function	0.38	0.01	0.08	0.02	0.23	0.80	0.07	0.86
Sptan1	333.20	Neurogenesis	0.02	0.00	0.01	0.00	0.01	0.12	0.00	0.98
App	2199.20	Serotonergic Synapse	0.10	0.00	0.03	0.00	0.05	0.05	0.00	0.94
Ms4a4a	214.00	Microglia Markers	0.13	0.01	0.97	0.11	0.64	0.12	0.02	0.66
Il10	76.02	Microglia-M2	0.22	0.01	0.77	0.08	0.67	0.16	0.03	0.89
Map1lc3b	621.03	Autophagy	0.68	0.00	0.60	0.04	0.90	0.11	0.04	1.00
Becn1	314.86	Autophagy	0.57	0.01	0.90	0.09	0.95	0.12	0.06	0.99
Gfap	138.41	Astrocyte Markers	0.90	0.00	0.84	0.02	0.99	0.03	0.02	1.00
Ywhaq	1291.06	Cell Cycle	0.27	0.00	0.51	0.01	0.57	0.02	0.00	0.98
C4a/b	269.28	Astrocyte Markers	0.88	0.00	0.46	0.00	0.91	0.00	0.00	0.97
Epb41l3	161.17	Cytoskeletal Dynamics	0.37	0.00	0.43	0.00	0.61	0.00	0.00	1.00
Arpc1a	576.49	Phagocytosis	0.50	0.00	0.93	0.00	0.94	0.00	0.00	0.97
Bax	192.11	Apoptosis	0.40	0.00	0.21	0.14	0.43	0.01	0.09	0.99
Cacna2d3	67.73	Ion Transport	0.30	0.00	0.51	0.01	0.60	0.00	0.02	0.99
Stat1	480.60	Interferon Signaling	0.71	0.00	0.47	0.09	0.85	0.02	0.06	0.99
Scn8a	95.43	Myelogenesis	0.92	0.00	0.38	0.07	0.94	0.01	0.02	0.88
H2-T23	883.41	Antigen Processing and Presentation	0.44	0.00	0.81	0.02	0.87	0.01	0.03	0.98
B2m	35445.12	Microglia-DAM Stage 1	0.24	0.00	0.37	0.00	1.00	0.00	0.01	0.45
Tyrobp	17491.26	Microglia-DAM Stage 1	0.87	0.00	0.49	0.01	0.98	0.03	0.02	0.92
C1qa	36650.02	Microglia-Homeostatic	0.61	0.00	0.60	0.01	1.00	0.03	0.03	0.87
C1qb	27676.80	Microglia-Homeostatic	0.64	0.00	0.58	0.02	1.00	0.08	0.07	0.87
Ppt1	1931.50	Lipid Metabolism	0.42	0.00	0.39	0.01	1.00	0.08	0.07	0.62
Atp6v1g1	530.88	Phagocytosis	0.79	0.00	0.75	0.02	0.97	0.03	0.02	1.00
Tspo	234.96	Microglia-M1	0.49	0.00	0.15	0.00	0.42	0.04	0.00	0.93
Fcgr4	296.67	Phagocytosis	0.42	0.00	0.06	0.00	0.78	0.02	0.01	0.22
Lamp2	4362.37	Autophagy	0.09	0.00	0.61	0.00	0.77	0.00	0.01	0.37
Grn	5765.84	Microglia-DAM Stage 1	0.05	0.00	0.95	0.00	0.41	0.00	0.01	0.46
Ctsl	1985.32	Microglia-DAM Stage 2-MGnD	0.03	0.00	0.54	0.00	0.51	0.00	0.00	0.17
Cd84	2961.69	Primed Microglia	0.02	0.00	0.67	0.00	0.41	0.00	0.00	0.18
Srgn	4008.85	A1 Astrocyte	0.36	0.00	0.59	0.00	0.71	0.01	0.00	0.99
Cd14	6633.99	A2 Astrocyte	0.91	0.00	0.51	0.00	0.98	0.00	0.00	0.94
Il1b	7758.06	Microglia-M1	0.77	0.00	0.78	0.00	0.97	0.00	0.00	1.00
Csf1	1774.01	Microglia-DAM Stage 2-MGnD	0.81	0.00	0.53	0.00	0.91	0.00	0.00	0.99
Tlr2	1855.79	Primed Microglia	0.24	0.00	0.55	0.00	0.97	0.00	0.00	0.57
H2-D1	4509.76	Antigen Processing and Presentation	0.75	0.00	0.61	0.00	1.00	0.00	0.00	0.93
Lamp1	4285.20	Autophagy	0.07	0.00	0.80	0.00	0.59	0.00	0.00	0.40
Ctsd	98930.23	Microglia-DAM Stage 1	0.52	0.00	0.70	0.00	1.00	0.00	0.00	0.87
Timp2	6859.80	Microglia-DAM Stage 1	0.25	0.00	0.56	0.00	0.97	0.00	0.00	0.58
Ctsb	12402.21	Microglia-DAM Stage 1	0.13	0.00	0.22	0.00	0.99	0.00	0.00	0.23
Gla	195.47	NO Metabolism and Signaling	0.35	0.00	0.21	0.00	0.99	0.00	0.00	0.40
Fabp5	125.74	Microglia-DAM Stage 1	0.91	0.00	0.93	0.00	1.00	0.00	0.00	1.00
Cst7	1880.34	Microglia-DAM Stage 2-MGnD	0.63	0.00	0.61	0.00	1.00	0.00	0.00	0.88
Spp1	6373.26	Microglia-DAM Stage 2-MGnD	0.92	0.00	0.88	0.00	1.00	0.00	0.00	1.00
Cd68	7056.76	Microglia-DAM Stage 2-MGnD	0.84	0.00	0.38	0.00	0.85	0.00	0.00	0.95
Itgax	263.83	Microglia-DAM Stage 2-MGnD	0.68	0.00	0.75	0.00	1.00	0.00	0.00	0.95
Lpl	570.95	Microglia-DAM Stage 2-MGnD	0.60	0.00	0.60	0.00	1.00	0.00	0.00	0.87
Apoe	39490.38	Microglia-DAM Stage 1	0.98	0.00	0.80	0.00	1.00	0.00	0.00	1.00
Axl	941.91	Microglia-DAM Stage 2-MGnD	0.90	0.00	0.78	0.00	1.00	0.00	0.00	0.99
Clec7a	2266.04	Microglia-DAM Stage 2-MGnD	0.83	0.00	0.61	0.00	1.00	0.00	0.00	0.95
Lgals3	1566.33	Microglia-DAM Stage 2-MGnD	0.87	0.00	0.43	0.00	0.96	0.00	0.00	0.89
Ptger4	197.48	Neuroactive Ligands and Receptors	0.50	0.00	0.95	0.00	0.95	0.00	0.00	0.97
Igf1	85.18	Microglia-M2	0.67	0.00	0.60	0.00	0.90	0.00	0.00	1.00
Fth1	50481.79	Microglia-DAM Stage 1	0.89	0.00	0.50	0.00	0.98	0.00	0.00	0.93
Lyz2	7058.21	Microglia-DAM Stage 1	0.82	0.00	0.39	0.00	0.96	0.00	0.00	0.85
Cybb	901.48	Microglia-M1	0.87	0.00	0.34	0.00	0.84	0.00	0.00	0.93
Atp6v1c1	1262.17	Primed Microglia	0.22	0.00	0.10	0.00	0.19	0.00	0.00	0.98
Ass1	84.87	NO Metabolism and Signaling	0.24	0.00	0.82	0.00	0.88	0.00	0.00	0.71
Ptprc	1208.19	Microglia-M1	0.55	0.00	0.90	0.00	0.98	0.00	0.00	0.95
Got1	511.97	Glutamatergic Synapse	0.19	0.00	0.12	0.00	1.00	0.00	0.00	0.19
Gpnmb	2840.49	Microglia-DAM Stage 1	0.40	0.00	0.43	0.00	1.00	0.00	0.00	0.63
Msr1	84.55	Microglia-DAM Stage 2-MGnD	0.06	0.00	0.15	0.00	0.97	0.00	0.00	0.10
C3	243.27	Primed Microglia	0.07	0.00	0.31	0.00	0.88	0.00	0.00	0.19
Pgk1	1433.26	Glucose Metabolism	0.07	0.00	0.04	0.00	0.05	0.00	0.00	0.99
Lilrb4a	1285.56	Microglia-DAM Stage 2-MGnD	0.11	0.00	0.50	0.00	0.86	0.00	0.00	0.35
Myo5a	302.69	Myelogenesis	0.83	0.00	0.90	0.00	0.99	0.00	0.00	1.00
Ptgs2	496.30	Microglia-M1	0.14	0.00	0.88	0.00	0.61	0.00	0.00	0.73
Aplp2	1314.62	Microglia-DAM Stage 1	0.28	0.00	0.33	0.00	0.45	0.00	0.00	1.00
Hspa9	927.18	Primed Microglia	0.22	0.00	0.81	0.00	0.69	0.00	0.00	0.87
Vim	943.56	Macrophage Function	0.31	0.00	0.33	0.00	0.48	0.00	0.00	1.00
Bhlhe40	343.24	Neuronal Markers	0.62	0.00	0.31	0.00	0.68	0.00	0.00	0.98
Ctnnb1	2100.69	Wnt Signaling	0.47	0.00	0.17	0.00	0.42	0.00	0.00	0.95
Ifnar1	735.89	Interferon Signaling	0.96	0.00	1.00	0.00	1.00	0.00	0.00	1.00
Ank	452.29	Primed Microglia	0.89	0.00	0.72	0.00	0.98	0.00	0.00	1.00
Atp6v1a	634.69	Primed Microglia	0.64	0.00	0.51	0.00	0.84	0.00	0.00	1.00
Gas7	107.64	Primed Microglia	0.66	0.00	0.53	0.00	0.86	0.00	0.00	1.00
Txn1	1284.32	Oxidative & Nitrosative Stress	0.43	0.00	0.51	0.00	0.71	0.00	0.00	1.00
Atp6v1b2	801.94	Primed Microglia	0.60	0.00	0.38	0.00	0.99	0.00	0.00	0.73
Cspg4	111.95	Neurogenesis	0.55	0.00	0.89	0.00	0.99	0.00	0.00	0.94
Atp6v0d1	893.27	Phagocytosis	0.86	0.00	0.77	0.00	0.99	0.00	0.00	1.00
Galc	320.65	Primed Microglia	0.32	0.00	0.20	0.00	1.00	0.00	0.00	0.37
Psmb8	894.83	Primed Microglia	0.88	0.00	0.57	0.00	0.95	0.00	0.00	0.99
Grb2	948.94	T-cell signaling	0.10	0.00	0.40	0.00	0.91	0.00	0.00	0.29
Gnas	1864.08	Serotonergic Synapse	0.64	0.00	0.62	0.01	1.00	0.00	0.00	0.89
Hacd2	225.77	Lipid Metabolism	0.63	0.00	0.77	0.00	0.94	0.00	0.00	1.00
Atp1a1	1181.52	Primed Microglia	0.94	0.00	0.19	0.00	0.78	0.02	0.01	0.72
Meaf6	178.59	Neurogenesis	0.98	0.00	0.93	0.04	1.00	0.05	0.04	1.00
Atp6v1d	492.55	Phagocytosis	0.24	0.00	0.25	0.00	0.36	0.02	0.00	1.00
Cd74	4450.02	Microglia-M1	0.69	0.01	0.41	0.07	0.80	0.36	0.10	0.99
Por	249.47	NO Metabolism and Signaling	0.52	0.00	0.16	0.01	0.92	0.24	0.10	0.44
Tomm20	849.69	Autophagy	0.88	0.00	0.97	0.04	1.00	0.04	0.03	1.00
Pgam1	552.02	Glucose Metabolism	0.90	0.00	0.63	0.03	0.97	0.07	0.04	0.99
Alas1	376.40	Primed Microglia	0.75	0.00	0.69	0.03	1.00	0.01	0.01	0.95
Ccl6	612.15	Microglia-DAM Stage 2-MGnD	0.73	0.01	0.84	0.13	0.98	0.20	0.11	1.00
Trem2	5474.90	Microglia-DAM Stage 2-MGnD	0.82	0.01	0.97	0.19	1.00	0.18	0.23	1.00
C1qc	28034.06	Microglia-Homeostatic	0.63	0.02	0.92	0.21	0.99	0.25	0.36	0.97
Syngr1	954.10	Neurogenesis	0.53	0.02	0.88	0.19	0.94	0.25	0.11	0.98
Cd9	6707.48	Microglia-DAM Stage 2-MGnD	0.03	0.00	0.87	0.00	0.27	0.00	0.04	0.37
Tap1	424.16	Primed Microglia	0.49	0.02	0.77	0.31	0.89	0.18	0.44	0.99
Gpr84	963.05	Microglia-M1	0.86	0.04	0.42	0.66	0.96	0.16	0.30	0.88
Pmp22	1740.30	Myelogenesis	0.53	0.02	0.57	0.47	1.00	0.16	0.15	0.81
Rtn4	1824.24	Neurogenesis	0.68	0.01	0.99	0.11	0.99	0.11	0.07	0.99
Csf3r	1682.53	Cytokines	0.00	0.00	0.53	0.03	0.12	0.01	0.00	0.03
Hmox1	902.84	Primed Microglia	0.04	0.00	0.78	0.07	0.48	0.04	0.01	0.30
Hsp90ab1	3385.72	Proteotoxic Stress	0.02	0.00	0.12	0.01	0.05	0.27	0.00	0.83
Stat2	139.11	Interferon Signaling	0.08	0.02	0.02	1.00	0.91	0.02	0.04	0.03
Adam22	53.84	Myelogenesis	0.34	0.01	0.18	0.55	0.99	0.04	0.06	0.36
Abi2	125.92	Neurogenesis	0.44	0.03	0.64	0.47	1.00	0.20	0.15	0.80
Gsk3b	479.12	Wnt Signaling	0.64	0.02	0.77	0.32	1.00	0.18	0.15	0.94
Dnaja2	281.93	Proteotoxic Stress	0.25	0.02	0.51	0.43	0.98	0.12	0.07	0.55
Tgfb1	2776.22	Microglia-Homeostatic	0.09	0.00	0.00	1.00	0.01	0.00	0.55	0.43
Arpc2	2046.01	Phagocytosis	0.29	0.02	0.11	0.86	0.25	0.04	0.62	0.97
Ywhag	722.72	Neurogenesis	0.84	0.01	0.10	0.60	0.50	0.02	0.14	0.66

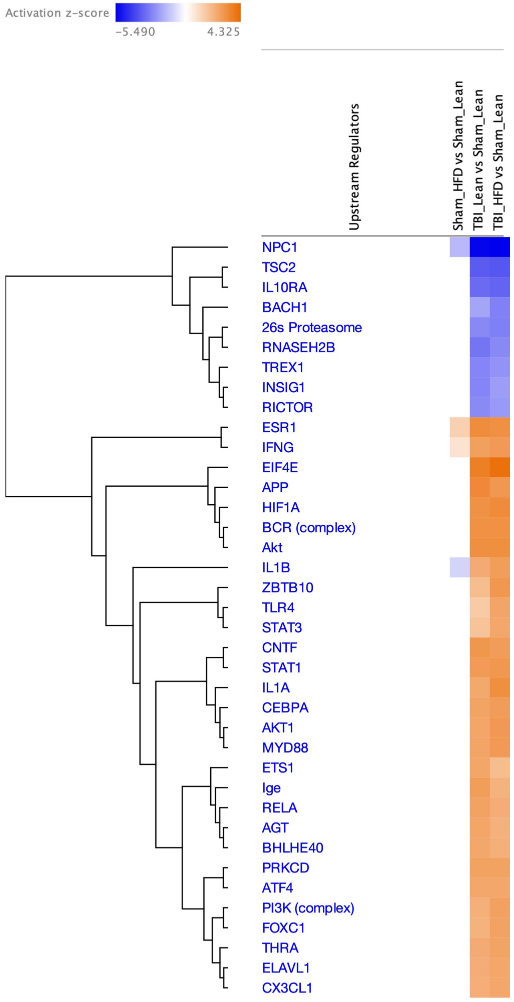

Upstream regulators – Genes (upregulated)

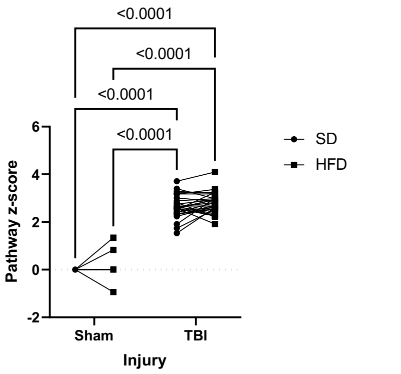

Source of Variation	% of total variation	P value	P value summary	Significant?	
Injury	93.23	<0.0001	****	Yes	
Diet	0.1260	0.0477	*	Yes	
Injury x Diet	0.04093	0.3049	ns	No	
Pathways x Injury	1.961				
Pathways x Diet	0.8223				
Pathways	2.771				
ANOVA table	SS	DF	MS	F (DFn, DFd)	P value
Injury	210.5	1	210.5	F (1, 28) = 1331	P<0.0001
Diet	0.2844	1	0.2844	F (1, 28) = 4.290	P=0.0477
Injury x Diet	0.09241	1	0.09241	F (1, 28) = 1.092	P=0.3049

Upstream regulators – Genes (downregulated)

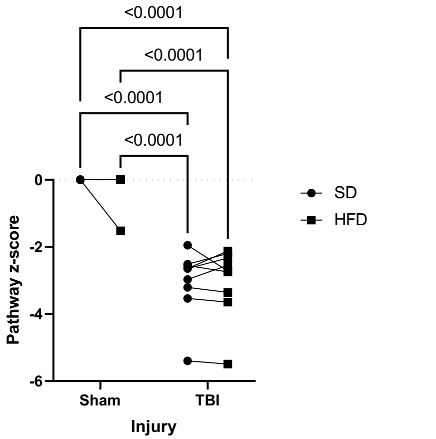

Source of Variation	% of total variation	P value	P value summary	Significant?	
Injury	80.49	<0.0001	****	Yes	
Diet	0.04470	0.5596	ns	No	
Injury x Diet	0.09182	0.3585	ns	No	
Pathways x Injury	5.046				
Pathways x Diet	0.9651				
Pathways	12.59				
ANOVA table	SS	DF	MS	F (DFn, DFd)	P value
Injury	78.28	1	78.28	F (1, 8) = 127.6	P<0.0001
Diet	0.04347	1	0.04347	F (1, 8) = 0.3705	P=0.5596
Injury x Diet	0.08930	1	0.08930	F (1, 8) = 0.9491	P=0.3585

Figure 8-Supplemental

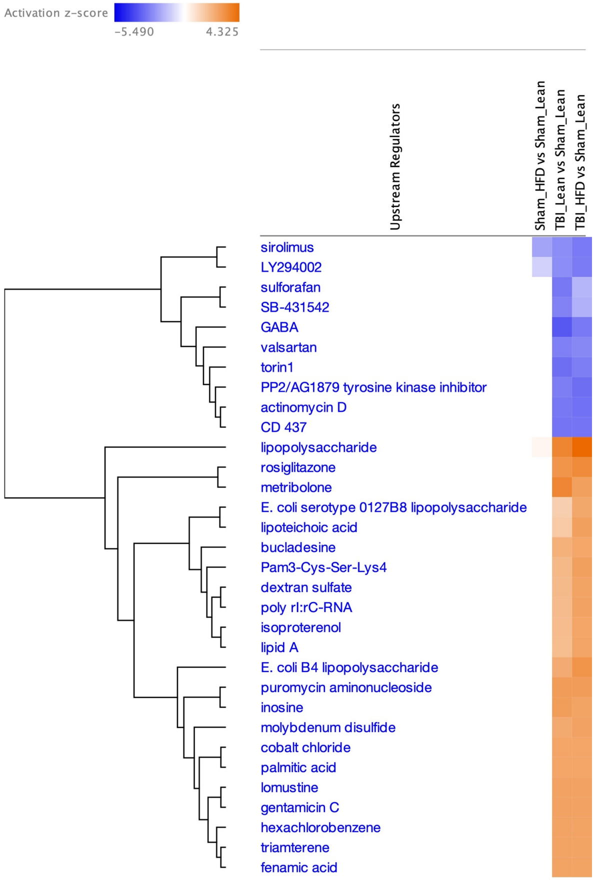

Upstream regulators – Drugs (upregulated)

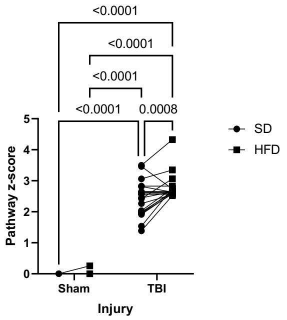

Source of Variation	% of total variation	P value	P value summary	Significant?	
Injury	93.38	<0.0001	****	Yes	
Diet	0.3788	0.0043	**	Yes	
Injury x Diet	0.3277	0.0049	**	Yes	
Pathways x Injury	2.013				
Pathways x Diet	0.7758				
Pathways	2.429				
ANOVA table	SS	DF	MS	F (DFn, DFd)	P value
Injury	149.1	1	149.1	F (1, 21) = 974.0	P<0.0001
Diet	0.6051	1	0.6051	F (1, 21) = 10.25	P=0.0043
Injury x Diet	0.5234	1	0.5234	F (1, 21) = 9.909	P=0.0049

Upstream regulators – Drugs (downregulated)

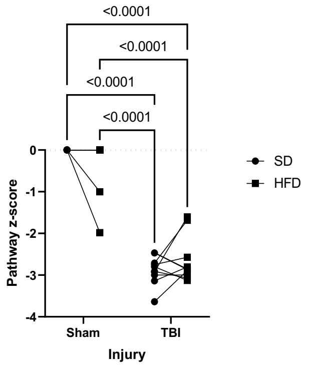

Source of Variation	% of total variation	P value	P value summary	Significant?	
Diet	88.90	<0.0001	****	Yes	
Injury	0.01425	0.8553	ns	No	
Diet x Injury	0.9141	0.0278	*	Yes	
Pathway x Diet	3.051				
Pathway x Injury	3.643				
Pathway	2.283				
ANOVA table	SS	DF	MS	F (DFn, DFd)	P value
Diet	68.35	1	68.35	F (1, 9) = 262.2	P<0.0001
Injury	0.01096	1	0.01096	F (1, 9) = 0.03521	P=0.8553
Diet x Injury	0.7028	1	0.7028	F (1, 9) = 6.860	P=0.0278

Figure 9-Supplemental

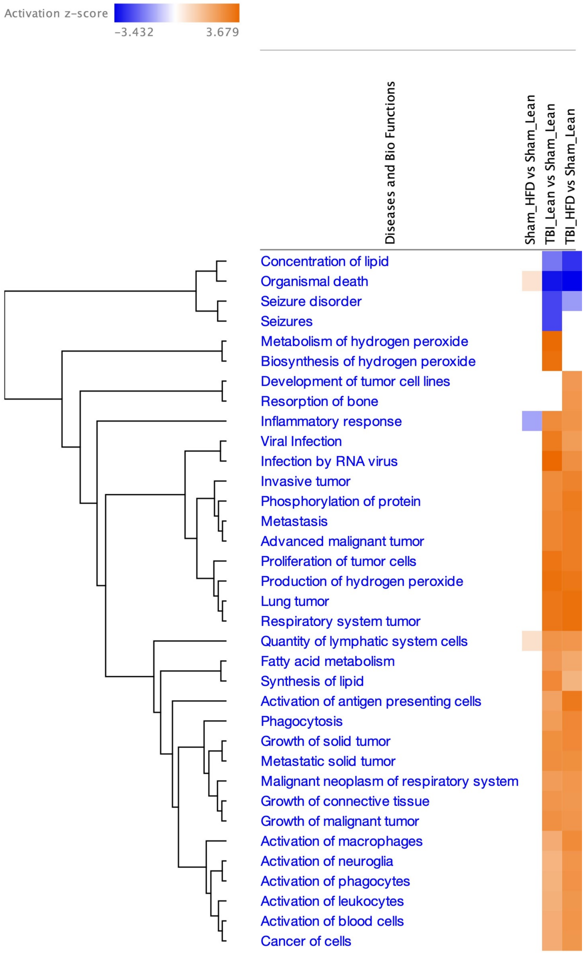

Diseases (upregulated)

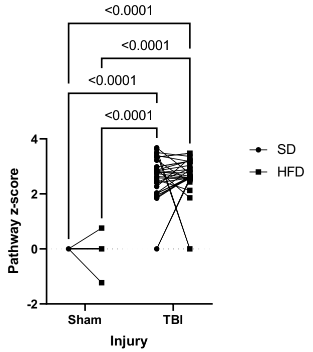

Source of Variation	% of total variation	P value	P value summary	Significant?	
Injury	82.76	<0.0001	****	Yes	
Diet	0.001747	0.9169	ns	No	
Injury x Diet	0.008928	0.8121	ns	No	
Pathway x Injury	3.984				
Pathway x Diet	4.737				
Pathway	3.853				
ANOVA table	SS	DF	MS	F (DFn, DFd)	P value
Injury	209.7	1	209.7	F (1, 30) = 623.2	P<0.0001
Diet	0.004428	1	0.004428	F (1, 30) = 0.01107	P=0.9169
Injury x Diet	0.02263	1	0.02263	F (1, 30) = 0.05752	P=0.8121

Diseases (downregulated)

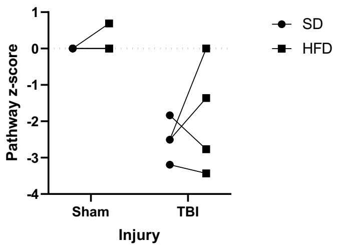

Source of Variation	% of total variation	P value	P value summary	Significant?	
Diet	2.092	0.3492	ns	No	
Injury	69.67	0.0203	*	Yes	
Diet x Injury	0.6655	0.6327	ns	No	
Pathway x Diet	5.123				
Pathway x Injury	10.23				
Pathway	5.118				
ANOVA table	SS	DF	MS	F (DFn, DFd)	P value
Diet	0.6281	1	0.6281	F (1, 3) = 1.225	P=0.3492
Injury	20.92	1	20.92	F (1, 3) = 20.42	P=0.0203
Diet x Injury	0.1998	1	0.1998	F (1, 3) = 0.2811	P=0.6327

Figure 10-Supplemental

Figure 11-Supplemental

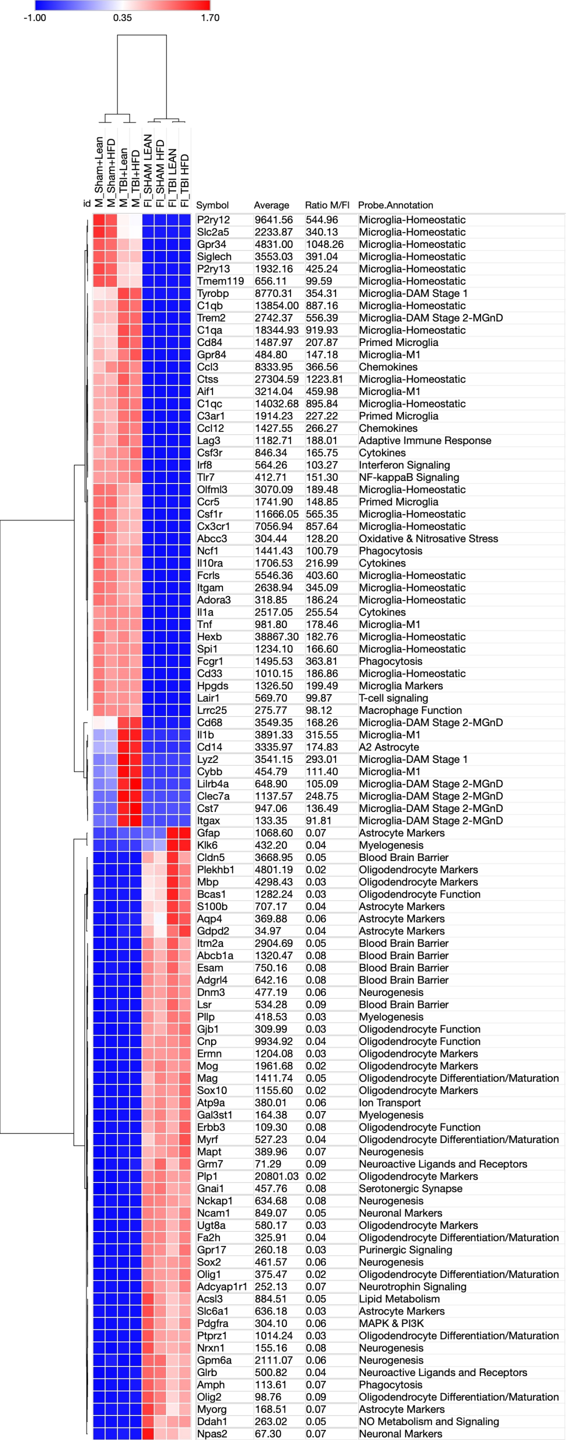

Symbol	Average	Ratio M/FI	Probe.Annotation
P2ry12	9641.56	544.96	Microglia-Homeostatic
Slc2a5	2233.87	340.13	Microglia-Homeostatic
Gpr34	4831.00	1048.26	Microglia-Homeostatic
Siglech	3553.03	391.04	Microglia-Homeostatic
P2ry13	1932.16	425.24	Microglia-Homeostatic
Tmem119	656.11	99.59	Microglia-Homeostatic
Tyrobp	8770.31	354.31	Microglia-DAM Stage 1
C1qb	13854.00	887.16	Microglia-Homeostatic
Trem2	2742.37	556.39	Microglia-DAM Stage 2-MGnD
C1qa	18344.93	919.93	Microglia-Homeostatic
Cd84	1487.97	207.87	Primed Microglia
Gpr84	484.80	147.18	Microglia-M1
Ccl3	8333.95	366.56	Chemokines
Ctss	27304.59	1223.81	Microglia-Homeostatic
Aif1	3214.04	459.98	Microglia-M1
C1qc	14032.68	895.84	Microglia-Homeostatic
C3ar1	1914.23	227.22	Primed Microglia
Ccl12	1427.55	266.27	Chemokines
Lag3	1182.71	188.01	Adaptive Immune Response
Csf3r	846.34	165.75	Cytokines
Irf8	564.26	103.27	Interferon Signaling
Tlr7	412.71	151.30	NF-kappaB Signaling
Olfml3	3070.09	189.48	Microglia-Homeostatic
Ccr5	1741.90	148.85	Primed Microglia
Csf1r	11666.05	565.35	Microglia-Homeostatic
Cx3cr1	7056.94	857.64	Microglia-Homeostatic
Abcc3	304.44	128.20	Oxidative & Nitrosative Stress
Ncf1	1441.43	100.79	Phagocytosis
Il10ra	1706.53	216.99	Cytokines
Fcrls	5546.36	403.60	Microglia-Homeostatic
Itgam	2638.94	345.09	Microglia-Homeostatic
Adora3	318.85	186.24	Microglia-Homeostatic
Il1a	2517.05	255.54	Cytokines
Tnf	981.80	178.46	Microglia-M1
Hexb	38867.30	182.76	Microglia-Homeostatic
Spi1	1234.10	166.60	Microglia-Homeostatic
Fcgr1	1495.53	363.81	Phagocytosis
Cd33	1010.15	186.86	Microglia-Homeostatic
Hpgds	1326.50	199.49	Microglia Markers
Lair1	569.70	99.87	T-cell signaling
Lrrc25	275.77	98.12	Macrophage Function
Cd68	3549.35	168.26	Microglia-DAM Stage 2-MGnD
Il1b	3891.33	315.55	Microglia-M1
Cd14	3335.97	174.83	A2 Astrocyte
Lyz2	3541.15	293.01	Microglia-DAM Stage 1
Cybb	454.79	111.40	Microglia-M1
Lilrb4a	648.90	105.09	Microglia-DAM Stage 2-MGnD
Clec7a	1137.57	248.75	Microglia-DAM Stage 2-MGnD
Cst7	947.06	136.49	Microglia-DAM Stage 2-MGnD
Itgax	133.35	91.81	Microglia-DAM Stage 2-MGnD
Gfap	1068.60	0.07	Astrocyte Markers
Klk6	432.20	0.04	Myelogenesis
Cldn5	3668.95	0.05	Blood Brain Barrier
Plekhb1	4801.19	0.02	Oligodendrocyte Markers
Mbp	4298.43	0.03	Oligodendrocyte Markers
Bcas1	1282.24	0.03	Oligodendrocyte Function
S100b	707.17	0.04	Astrocyte Markers
Aqp4	369.88	0.06	Astrocyte Markers
Gdpd2	34.97	0.04	Astrocyte Markers
Itm2a	2904.69	0.05	Blood Brain Barrier
Abcb1a	1320.47	0.08	Blood Brain Barrier
Esam	750.16	0.08	Blood Brain Barrier
Adgrl4	642.16	0.08	Blood Brain Barrier
Dnm3	477.19	0.06	Neurogenesis
Lsr	534.28	0.09	Blood Brain Barrier
Pllp	418.53	0.03	Myelogenesis
Gjb1	309.99	0.03	Oligodendrocyte Function
Cnp	9934.92	0.04	Oligodendrocyte Function
Ermn	1204.08	0.03	Oligodendrocyte Markers
Mog	1961.68	0.02	Oligodendrocyte Markers
Mag	1411.74	0.05	Oligodendrocyte Differentiation/Maturation
Sox10	1155.60	0.02	Oligodendrocyte Markers
Atp9a	380.01	0.06	Ion Transport
Gal3st1	164.38	0.07	Myelogenesis
Erbb3	109.30	0.08	Oligodendrocyte Function
Myrf	527.23	0.04	Oligodendrocyte Differentiation/Maturation
Mapt	389.96	0.07	Neurogenesis
Grm7	71.29	0.09	Neuroactive Ligands and Receptors
Plp1	20801.03	0.02	Oligodendrocyte Markers
Gnai1	457.76	0.08	Serotonergic Synapse
Nckap1	634.68	0.08	Neurogenesis
Ncam1	849.07	0.05	Neuronal Markers
Ugt8a	580.17	0.03	Oligodendrocyte Markers
Fa2h	325.91	0.04	Oligodendrocyte Differentiation/Maturation
Gpr17	260.18	0.03	Purinergic Signaling
Sox2	461.57	0.06	Neurogenesis
Olig1	375.47	0.02	Oligodendrocyte Differentiation/Maturation
Adcyap1r1	252.13	0.07	Neurotrophin Signaling
Acsl3	884.51	0.05	Lipid Metabolism
Slc6a1	636.18	0.03	Astrocyte Markers
Pdgfra	304.10	0.06	MAPK & PI3K
Ptprz1	1014.24	0.03	Oligodendrocyte Differentiation/Maturation
Nrxn1	155.16	0.08	Neurogenesis
Gpm6a	2111.07	0.06	Neurogenesis
Glrb	500.82	0.04	Neuroactive Ligands and Receptors
Amph	113.61	0.07	Phagocytosis
Olig2	98.76	0.09	Oligodendrocyte Differentiation/Maturation
Myorg	168.51	0.07	Astrocyte Markers
Ddah1	263.02	0.05	NO Metabolism and Signaling
Npas2	67.30	0.07	Neuronal Markers

Figure 12-Supplemental

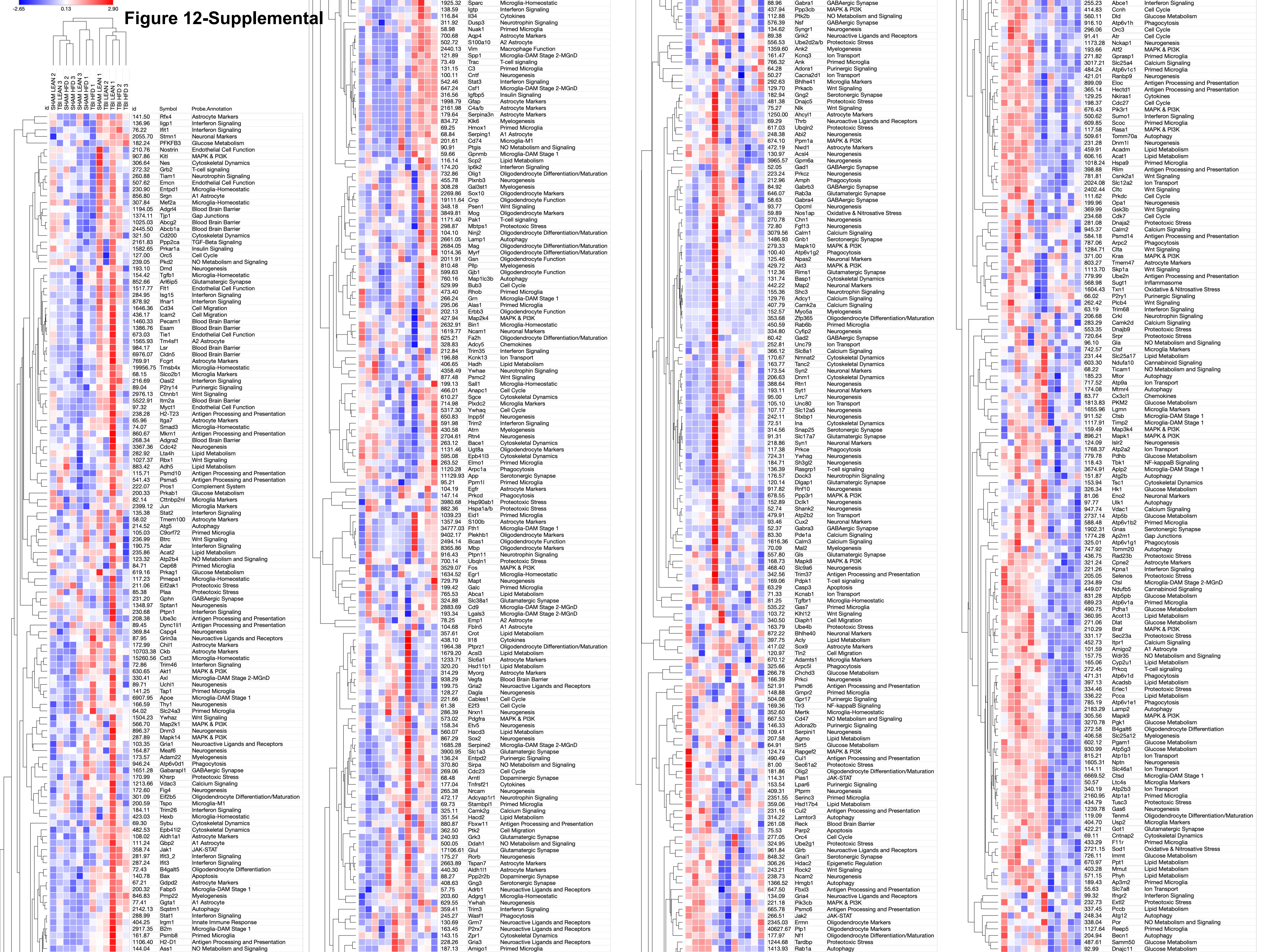

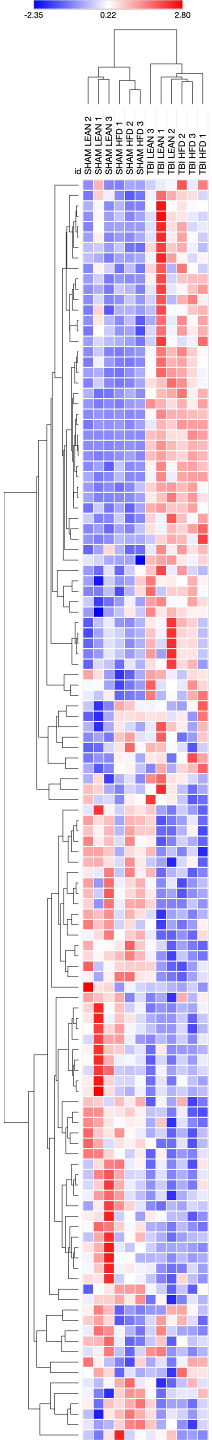

Symbol	Average	Probe.Annotation	p_val_Diet	p_val_Condition	p_val_diet:condition	p_TBILean:ShamLean	p_TBIHFD:ShamLean	p_TBIHFD:ShamHFD	p_ShamHFD:ShamLean	p_TBIHFD:TBILean
Egfr	104.19	Astrocyte Markers	0.46	0.04	0.09	0.97	0.17	0.06	0.85	0.30
Aldh1a1	108.02	Astrocyte Markers	0.06	0.01	0.57	0.28	0.84	0.09	0.28	0.69
H2-D1	1106.40	Antigen Processing and Presentation	0.23	0.02	0.74	0.16	0.62	0.30	0.91	0.67
B2m	2917.35	Microglia-DAM Stage 1	0.25	0.04	0.46	0.20	0.85	0.68	0.99	0.52
Irgm1	404.25	Innate Immune Response	0.38	0.03	0.68	0.20	0.61	0.42	0.98	0.77
Stat1	288.99	Interferon Signaling	0.39	0.03	0.81	0.26	0.66	0.41	0.96	0.84
Igtp	138.59	Interferon Signaling	0.10	0.00	0.48	0.05	0.54	0.20	0.85	0.32
Sparc	1925.32	Microglia-Homeostatic	0.18	0.00	0.17	0.02	0.39	0.40	1.00	0.23
Jak1	358.74	JAK-STAT	0.45	0.04	0.66	0.56	0.69	0.26	0.81	0.99
Ggta1	77.41	A1 Astrocyte	0.94	0.00	0.71	0.09	0.07	0.04	0.99	1.00
Sqstm1	2142.13	Autophagy	0.66	0.01	0.86	0.11	0.08	0.15	0.97	1.00
Pmp22	846.83	Myelogenesis	0.53	0.02	0.57	0.36	0.11	0.12	1.00	0.82
B4galt5	72.43	Oligodendrocyte Differentiation	0.97	0.04	0.95	0.39	0.35	0.35	1.00	1.00
Gdpd2	67.21	Astrocyte Markers	0.93	0.03	0.25	0.74	0.32	0.09	0.78	0.84
Fabp5	200.32	Microglia-DAM Stage 1	0.75	0.01	0.24	0.41	0.17	0.04	0.68	0.91
Tap1	141.25	Primed Microglia	0.60	0.03	0.85	0.37	0.49	0.27	0.95	0.99
S100a10	502.72	A2 Astrocyte	0.68	0.00	0.82	0.02	0.02	0.01	0.97	1.00
Aqp4	700.68	Astrocyte Markers	0.25	0.00	0.87	0.02	0.07	0.02	0.76	0.87
Vim	2440.13	Macrophage Function	0.36	0.00	0.48	0.01	0.03	0.02	1.00	0.64
Spp1	121.89	Microglia-DAM Stage 2-MGnD	0.36	0.00	0.75	0.01	0.03	0.02	0.97	0.80
Serping1	68.84	A1 Astrocyte	0.41	0.00	0.91	0.01	0.00	0.00	0.95	0.90
Hmox1	69.25	Primed Microglia	0.98	0.00	0.56	0.00	0.00	0.00	0.97	0.98
Gfap	1998.79	Astrocyte Markers	0.99	0.00	0.96	0.00	0.00	0.00	1.00	1.00
Igfbp5	316.56	Insulin Signaling	0.04	0.00	0.12	0.00	0.00	0.00	0.96	0.07
C4a/b	2161.98	Astrocyte Markers	0.38	0.00	0.50	0.00	0.00	0.00	1.00	0.67
Serpina3n	179.64	Astrocyte Markers	0.63	0.00	0.39	0.00	0.00	0.00	0.99	0.75
Klk6	834.72	Myelogenesis	0.72	0.00	0.39	0.00	0.00	0.00	0.80	0.98
Stat3	542.46	Interferon Signaling	0.21	0.00	0.94	0.00	0.00	0.00	0.80	0.74
Csf1	647.24	Microglia-DAM Stage 2-MGnD	0.18	0.00	0.30	0.02	0.00	0.00	0.99	0.33
Cntf	100.11	Neurogenesis	0.77	0.00	0.45	0.00	0.00	0.00	0.98	0.86
C3	131.15	Primed Microglia	0.90	0.00	0.75	0.00	0.00	0.00	1.00	0.99
Trac	73.49	T-cell signaling	0.55	0.00	0.59	0.00	0.01	0.00	1.00	0.84
Gpnmb	59.66	Microglia-DAM Stage 1	0.76	0.00	0.90	0.05	0.07	0.06	1.00	0.99
Cd74	201.61	Microglia-M1	0.06	0.00	0.43	0.01	0.00	0.00	0.79	0.23
Ptgis	90.91	NO Metabolism and Signaling	0.24	0.00	0.80	0.10	0.02	0.06	0.89	0.71
Nuak1	58.98	Primed Microglia	0.74	0.01	0.31	0.29	0.14	0.03	0.75	0.95
Dusp3	311.92	Neurotrophin Signaling	0.02	0.00	0.70	0.04	0.40	0.02	0.19	0.39
Scp2	116.14	Lipid Metabolism	0.59	0.04	0.32	0.14	0.58	0.72	0.77	0.68
Slc38a1	324.88	Glutamatergic Synapse	0.57	0.02	0.27	0.07	0.13	0.61	0.61	0.97
Abca1	765.53	Lipid Metabolism	0.75	0.02	0.69	0.16	0.18	0.35	0.95	1.00
Cd9	2883.69	Microglia-DAM Stage 2-MGnD	0.66	0.00	0.22	0.01	0.03	0.19	0.60	0.92
Lgals3	193.34	Microglia-DAM Stage 2-MGnD	0.31	0.00	0.03	0.01	0.02	0.69	0.11	0.69
S100b	1357.94	Astrocyte Markers	0.47	0.02	0.70	0.14	0.41	0.31	0.99	0.84
Fth1	34777.03	Microglia-DAM Stage 1	0.55	0.02	0.24	0.06	0.38	0.62	0.97	0.56
Plekhb1	9402.17	Oligodendrocyte Markers	0.48	0.02	0.33	0.08	0.44	0.54	1.00	0.61
Mbp	8365.86	Oligodendrocyte Markers	0.55	0.05	0.42	0.19	0.64	0.73	1.00	0.73
Eid1	1039.23	Primed Microglia	0.48	0.03	0.47	0.17	0.59	0.60	1.00	0.72
Jun	2399.12	Microglia Markers	0.03	0.04	0.24	0.85	1.00	0.12	0.10	0.79
Fos	3529.07	MAPK & PI3K	0.23	0.01	0.22	0.56	0.56	0.05	0.32	1.00
Egr1	1634.52	Microglia-Homeostatic	0.37	0.04	0.13	0.93	0.69	0.07	0.32	0.95
Lgmn	1655.96	Microglia Markers	0.04	0.42	0.25	0.49	0.18	0.99	0.12	0.85
Ctsb	911.52	Microglia-DAM Stage 1	0.05	0.11	0.06	0.09	0.07	0.99	0.05	1.00
Hexb	423.03	Microglia-Homeostatic	0.85	0.04	0.12	0.07	0.30	0.95	0.55	0.71
Eif2b5	301.09	Oligodendrocyte Differentiation/Maturation	0.61	0.56	0.02	0.15	0.85	0.44	0.16	0.41
Clta	1284.71	Wnt Signaling	0.55	0.82	0.00	0.06	0.93	0.10	0.04	0.15
Myrf	1014.36	Oligodendrocyte Differentiation/Maturation	0.03	0.04	0.78	0.46	0.03	0.28	0.43	0.27
Gsn	2011.91	Oligodendrocyte Function	0.02	0.04	0.84	0.46	0.02	0.33	0.27	0.18
Psma5	541.43	Antigen Processing and Presentation	0.16	0.03	0.15	0.07	0.89	0.90	1.00	0.20
Ctnnb1	2976.13	Wnt Signaling	0.02	0.58	0.34	0.69	0.39	0.99	0.54	0.09
Plcb4	262.42	Wnt Signaling	0.04	0.91	0.10	0.62	0.35	0.53	0.98	0.07
Orc4	277.05	Cell Cycle	0.36	0.05	0.95	0.38	0.17	0.42	0.88	0.91
Hectd1	365.14	Antigen Processing and Presentation	0.61	0.02	0.65	0.18	0.17	0.42	0.89	1.00
Nkiras1	129.25	Cytokines	0.83	0.05	0.82	0.34	0.34	0.50	0.99	1.00
Cdc27	198.37	Cell Cycle	0.63	0.01	0.64	0.10	0.10	0.25	0.90	1.00
Ranbp9	421.01	Neurogenesis	0.07	0.02	0.97	0.28	0.03	0.26	0.49	0.46
Glrb	961.84	Neuroactive Ligands and Receptors	0.72	0.01	0.32	0.06	0.24	0.44	0.96	0.75
Slc25a4	3017.21	Calcium Signaling	0.47	0.05	0.68	0.57	0.21	0.29	1.00	0.84
Dlat	271.06	Glucose Metabolism	0.41	0.03	0.58	0.50	0.14	0.19	1.00	0.75
Acot13	360.95	Lipid Metabolism	0.34	0.01	0.68	0.22	0.05	0.10	0.97	0.74
Braf	210.29	MAPK & PI3K	0.98	0.04	0.59	0.57	0.36	0.22	0.98	0.97
Nwd1	472.19	Astrocyte Markers	0.51	0.00	0.22	0.01	0.02	0.13	0.51	0.97
Acsl4	130.97	Neurogenesis	0.25	0.02	0.13	0.04	0.06	0.78	0.23	0.99
Gpm6a	3965.57	Neurogenesis	0.75	0.01	0.63	0.11	0.24	0.28	1.00	0.93
Pdhb	779.78	Glucose Metabolism	0.94	0.00	0.16	0.29	0.06	0.01	0.73	0.67
Atp2a2	1768.37	Ion Transport	0.36	0.00	0.36	0.06	0.06	0.01	0.55	1.00
Kpna1	221.26	Interferon Signaling	0.91	0.04	0.67	0.51	0.33	0.25	1.00	0.98
Prkcq	272.45	T-cell signaling	0.23	0.01	0.12	0.76	0.58	0.04	0.22	0.99
Rapgef2	124.74	MAPK & PI3K	0.05	0.04	0.12	0.07	0.04	0.96	0.08	0.98
Acly	397.75	Lipid Metabolism	0.48	0.08	0.02	0.04	0.80	0.94	0.50	0.13
Myorg	314.29	Astrocyte Markers	0.76	0.01	0.08	0.02	0.10	0.71	0.42	0.66
Vegfa	938.29	Blood Brain Barrier	0.56	0.01	0.47	0.08	0.10	0.36	0.77	1.00
Gria2	199.75	Neuroactive Ligands and Receptors	0.46	0.01	0.59	0.09	0.07	0.26	0.78	1.00
Dagla	128.27	Neurogenesis	0.59	0.04	0.23	0.10	0.20	0.82	0.58	0.95
Hsd11b1	320.20	Lipid Metabolism	0.21	0.02	0.32	0.08	0.06	0.55	0.38	1.00
Slc6a1	1233.71	Astrocyte Markers	0.47	0.01	0.05	0.02	0.08	0.90	0.21	0.69
Rorb	175.27	Neurogenesis	0.15	0.05	0.05	0.05	0.10	1.00	0.10	0.96
Acsl3	1679.20	Lipid Metabolism	0.37	0.03	0.07	0.04	0.12	0.97	0.21	0.84
Ptprz1	1964.38	Oligodendrocyte Differentiation/Maturation	0.12	0.04	0.08	0.06	0.07	0.99	0.12	1.00
Sirt5	64.91	Glucose Metabolism	0.71	0.04	0.81	0.43	0.48	0.28	0.97	1.00
Mertk	352.60	Microglia-Homeostatic	0.20	0.00	0.84	0.03	0.01	0.05	0.69	0.84
Cd47	667.53	NO Metabolism and Signaling	0.33	0.02	0.58	0.13	0.09	0.39	0.68	0.99
Serpini1	109.41	Neurogenesis	0.45	0.00	0.62	0.04	0.03	0.11	0.79	1.00
Adora2b	146.33	Purinergic Signaling	0.69	0.01	0.61	0.10	0.12	0.29	0.91	1.00
Tlr3	169.36	NF-kappaB Signaling	0.21	0.01	0.13	0.02	0.03	0.49	0.22	1.00
Gabra4	58.63	GABAergic Synapse	0.71	0.04	0.82	0.31	0.52	0.46	1.00	0.97
Gabrb3	84.92	GABAergic Synapse	0.53	0.03	0.36	0.12	0.16	0.62	0.67	1.00
Rab3a	646.07	Glutamatergic Synapse	0.82	0.04	0.32	0.14	0.30	0.77	0.79	0.93
Amph	212.96	Phagocytosis	0.92	0.02	0.14	0.06	0.25	0.84	0.63	0.71
Opcml	93.77	Neurogenesis	0.59	0.03	0.65	0.19	0.48	0.45	1.00	0.88
Mapk8	168.73	MAPK & PI3K	0.02	0.06	0.63	0.30	0.03	0.65	0.16	0.39
Cables1	221.66	Cell Cycle	0.22	0.00	0.31	0.01	0.01	0.05	0.38	1.00
Ahcyl1	1250.00	Astrocyte Markers	0.54	0.03	0.34	0.13	0.18	0.68	0.66	0.99
Dnajc5	481.38	Proteotoxic Stress	0.04	0.00	0.11	0.01	0.01	0.44	0.07	0.95
Gng2	182.94	Serotonergic Synapse	0.14	0.01	0.12	0.03	0.03	0.70	0.15	1.00
Nlk	75.27	Wnt Signaling	0.32	0.01	0.29	0.06	0.06	0.49	0.46	1.00
Abi2	248.38	Neurogenesis	0.65	0.02	0.06	0.03	0.18	0.98	0.29	0.62
Mapk1	896.21	MAPK & PI3K	0.13	0.05	0.13	0.97	0.97	0.09	0.16	1.00
Islr2	124.09	Neurogenesis	0.31	0.01	0.93	0.14	0.35	0.11	0.83	0.89
Rfx4	141.50	Astrocyte Markers	0.12	0.72	0.02	0.31	0.77	0.15	0.04	0.80
Iigp1	136.96	Interferon Signaling	0.01	0.44	0.12	0.92	0.38	0.34	0.03	0.72
Ptk2	362.50	Cell Migration	0.01	0.28	0.63	0.65	0.06	0.96	0.12	0.31
Gad1	52.05	GABAergic Synapse	0.06	0.36	0.03	0.12	0.20	0.65	0.04	0.98
Plaa	85.38	Proteotoxic Stress	0.03	0.99	0.95	1.00	0.33	1.00	0.35	0.31
Bhlhe40	872.22	Neuronal Markers	0.86	0.83	0.04	0.43	0.99	0.29	0.41	0.30
Adora1	64.28	Purinergic Signaling	0.52	1.00	0.00	0.07	0.96	0.07	0.13	0.03
Atg2b	151.87	Autophagy	0.05	0.12	0.94	0.59	0.98	0.66	0.44	0.39
Gla	96.10	NO Metabolism and Signaling	0.01	0.75	0.14	0.80	0.21	0.55	0.03	0.60
B4galt6	272.58	Oligodendrocyte Differentiation	0.44	0.05	0.17	0.93	0.70	0.09	0.41	0.96
Ap3m2	189.43	Primed Microglia	0.12	0.29	0.04	0.81	0.97	0.14	0.08	0.96
F11r	433.29	Primed Microglia	0.44	0.05	0.00	0.66	0.73	0.01	0.04	0.20
Klhl12	103.72	Wnt Signaling	0.90	0.37	0.04	0.75	0.86	0.16	0.44	0.35

Figure 13-Supplemental

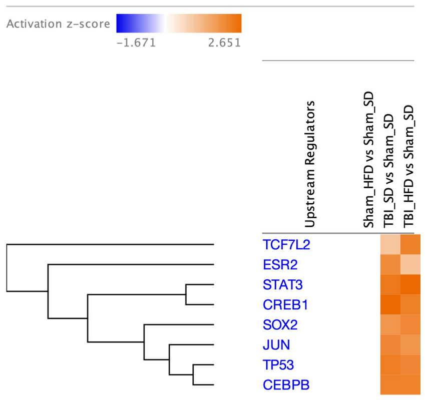

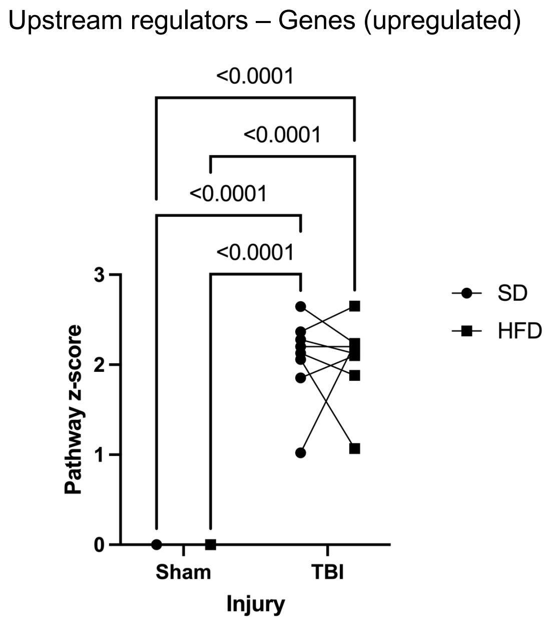

Source of Variation	% of total variation	P value	P value summary	Significant?	
Injury	91.72	<0.0001	****	Yes	
Diet	0.0002727	0.9757	ns	No	
Injury x Diet	0.0002727	0.9757	ns	No	
Subject x Injury	2.226				
Subject x Diet	1.913				
Subject	2.226				
ANOVA table	SS	DF	MS	F (DFn, DFd)	P value
Injury	34.15	1	34.15	F (1, 7) = 288.5	P<0.0001
Diet	0.0001015	1	0.0001015	F (1, 7) = 0.0009978	P=0.9757
Injury x Diet	0.0001015	1	0.0001015	F (1, 7) = 0.0009978	P=0.9757

Figure 14-Supplemental

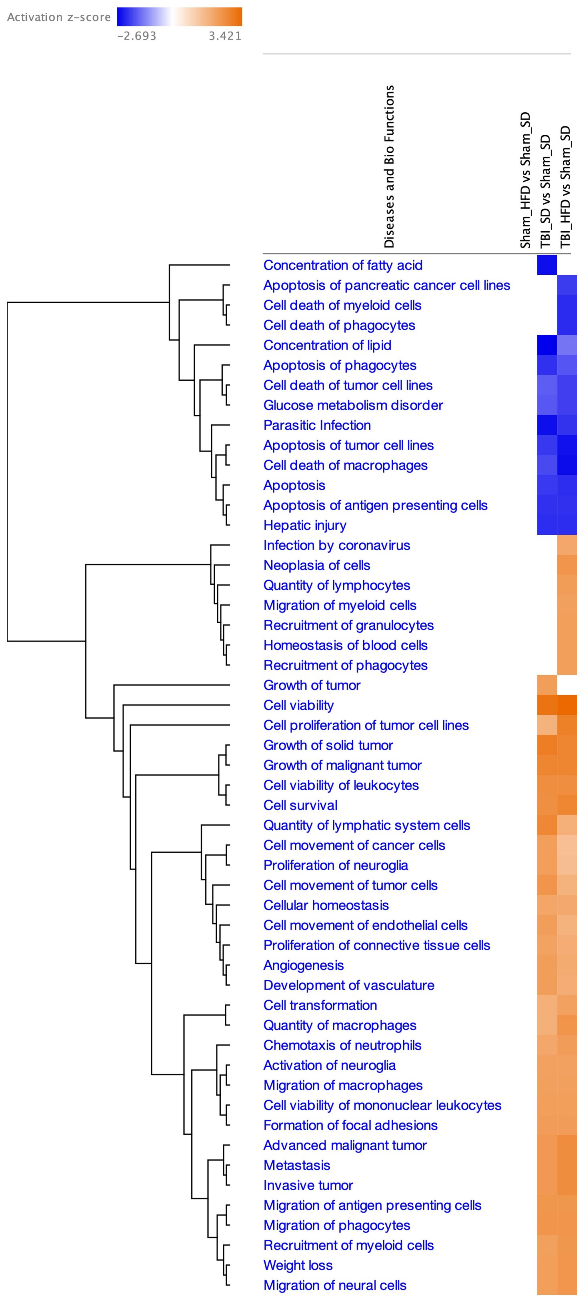

Diseases (upregulated)

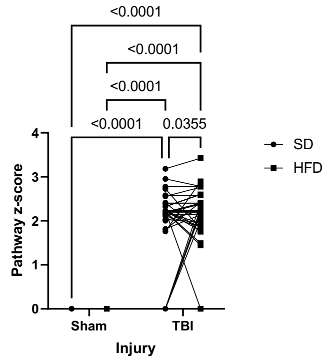

Source of Variation	% of total variation	P value	P value summary	Significant?	
Injury	77.53	<0.0001	****	Yes	
Diet	0.5334	0.0523	ns	No	
Injury x Diet	0.5334	0.0523	ns	No	
Pathway x Injury	5.792				
Pathway x Diet	4.909				
Pathway	5.792				
ANOVA table	SS	DF	MS	F (DFn, DFd)	P value
Injury	157.9	1	157.9	F (1, 37) = 495.3	P<0.0001
Diet	1.087	1	1.087	F (1, 37) = 4.020	P=0.0523
Injury x Diet	1.087	1	1.087	F (1, 37) = 4.020	P=0.0523

Diseases (downregulated)

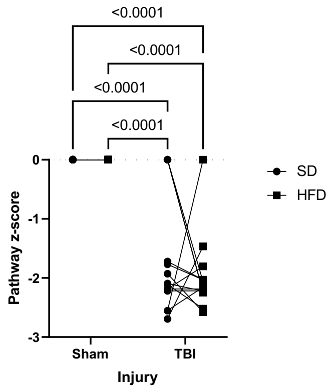

Source of Variation	% of total variation	P value	P value summary	Significant?	
Injury	72.75	<0.0001	****	Yes	
Diet	0.3818	0.4590	ns	No	
Injury x Diet	0.3818	0.4590	ns	No	
Pathway x Injury	4.722				
Pathway x Diet	8.522				
Pathway	4.722				
ANOVA table	SS	DF	MS	F (DFn, DFd)	P value
Injury	47.87	1	47.87	F (1, 13) = 200.3	P<0.0001
Diet	0.2513	1	0.2513	F (1, 13) = 0.5825	P=0.4590
Injury x Diet	0.2513	1	0.2513	F (1, 13) = 0.5825	P=0.4590

Figure 15-Supplemental

A Adipose Tissue – Fig. 4 & 5

Homeostatic Associated Macrophage

Two-way ANOVA	Ordinary				
Alpha	0.05				
Source of Variation	% of total variation	P value	P value summary	Significant?	
Interaction	9.361	0.0284	*	Yes	
Diet	73.80	<0.0001	****	Yes	
Injury	6.324	0.0596	ns	No	
ANOVA table	SS	DF	MS	F (DFn, DFd)	P value
Interaction	182.0	1	182.0	F (1, 8) = 7.123	P=0.0284
Diet	1435	1	1435	F (1, 8) = 56.16	P<0.0001
Injury	123.0	1	123.0	F (1, 8) = 4.813	P=0.0596
Residual	204.5	8	25.56		

Disease Associated Macrophage

Two-way ANOVA	Ordinary				
Alpha	0.05				
Source of Variation	% of total variation	P value	P value summary	Significant?	
Interaction	5.465	0.1190	ns	No	
Diet	70.91	0.0002	***	Yes	
Injury	9.276	0.0525	ns	No	
ANOVA table	SS	DF	MS	F (DFn, DFd)	P value
Interaction	105.7	1	105.7	F (1, 8) = 3.048	P=0.1190
Diet	1372	1	1372	F (1, 8) = 39.55	P=0.0002
Injury	179.4	1	179.4	F (1, 8) = 5.173	P=0.0525
Residual	277.5	8	34.69		

Macrophage Function

Two-way ANOVA	Ordinary				
Alpha	0.05				
Source of Variation	% of total variation	P value	P value summary	Significant?	
Interaction	7.680	0.0656	ns	No	
Diet	59.71	0.0003	***	Yes	
Injury	19.09	0.0099	**	Yes	
ANOVA table	SS	DF	MS	F (DFn, DFd)	P value
Interaction	1227	1	1227	F (1, 8) = 4.545	P=0.0656
Diet	9538	1	9538	F (1, 8) = 35.34	P=0.0003
Injury	3050	1	3050	F (1, 8) = 11.30	P=0.0099
Residual	2159	8	269.9		

Inflammatory Signaling

Two-way ANOVA	Ordinary				
Alpha	0.05				
Source of Variation	% of total variation	P value	P value summary	Significant?	
Interaction	3.029	0.3567	ns	No	
Diet	64.53	0.0020	**	Yes	
Injury	7.116	0.1722	ns	No	
ANOVA table	SS	DF	MS	F (DFn, DFd)	P value
Interaction	40.46	1	40.46	F (1, 8) = 0.9567	P=0.3567
Diet	861.9	1	861.9	F (1, 8) = 20.38	P=0.0020
Injury	95.05	1	95.05	F (1, 8) = 2.248	P=0.1722
Residual	338.3	8	42.29		

Innate Immunity

Two-way ANOVA	Ordinary				
Alpha	0.05				
Source of Variation	% of total variation	P value	P value summary	Significant?	
Interaction	17.87	0.0425	*	Yes	
Diet	12.69	0.0768	ns	No	
Injury	44.83	0.0051	**	Yes	
ANOVA table	SS	DF	MS	F (DFn, DFd)	P value
Interaction	807.4	1	807.4	F (1, 8) = 5.806	P=0.0425
Diet	573.5	1	573.5	F (1, 8) = 4.124	P=0.0768
Injury	2026	1	2026	F (1, 8) = 14.57	P=0.0051
Residual	1113	8	139.1		

Pro-inflammatory

Two-way ANOVA	Ordinary				
Alpha	0.05				
Source of Variation	% of total variation	P value	P value summary	Significant?	
Interaction	3.056	0.1129	ns	No	
Diet	82.03	<0.0001	****	Yes	
Injury	7.194	0.0258	*	Yes	
ANOVA table	SS	DF	MS	F (DFn, DFd)	P value
Interaction	7.134	1	7.134	F (1, 8) = 3.168	P=0.1129
Diet	191.5	1	191.5	F (1, 8) = 85.04	P<0.0001
Injury	16.79	1	16.79	F (1, 8) = 7.458	P=0.0258
Residual	18.01	8	2.252		

Canonical genes (upregulated)

Source of Variation	% of total variation	P value	P value summary	Significant?	
Diet	86.09	<0.0001	****	Yes	
Injury	2.593	<0.0001	****	Yes	
Diet x Injury	1.890	<0.0001	****	Yes	
Pathway x Diet	3.463				
Pathway x Injury	1.131				
Pathway	3.742				
ANOVA table	SS	DF	MS	F (DFn, DFd)	P value
Diet	348.6	1	348.6	F (1, 52) = 1293	P<0.0001
Injury	10.50	1	10.50	F (1, 52) = 119.2	P<0.0001
Diet x Injury	7.655	1	7.655	F (1, 52) = 90.09	P<0.0001
Pathway x Diet	14.02	52	0.2696		
Pathway x Injury	4.578	52	0.08805		
Pathway	15.15	52	0.2914		
Residual	4.418	52	0.08497		

B Microglia – Fig. 8 & 9

Homeostatic Associated Microglia

Two-way ANOVA	Ordinary				
Alpha	0.05				
Source of Variation	% of total variation	P value	P value summary	Significant?	
Interaction	1.591	0.3838	ns	No	
Diet	9.135	0.0583	ns	No	
Injury	74.28	0.0002	***	Yes	
ANOVA table	SS	DF	MS	F (DFn, DFd)	P value
Interaction	76.58	1	76.58	F (1, 8) = 0.8490	P=0.3838
Diet	439.6	1	439.6	F (1, 8) = 4.874	P=0.0583
Injury	3574	1	3574	F (1, 8) = 39.63	P=0.0002
Residual	721.6	8	90.20		

Autophagy Associated Microglia

Two-way ANOVA	Ordinary				
Alpha	0.05				
Source of Variation	% of total variation	P value	P value summary	Significant?	
Interaction	0.001490	0.9722	ns	No	
Diet	0.08478	0.7932	ns	No	
Injury	90.68	<0.0001	****	Yes	
ANOVA table	SS	DF	MS	F (DFn, DFd)	P value
Interaction	0.01219	1	0.01219	F (1, 8) = 0.001292	P=0.9722
Diet	0.6933	1	0.6933	F (1, 8) = 0.07348	P=0.7932
Injury	741.6	1	741.6	F (1, 8) = 78.60	P<0.0001
Residual	75.48	8	9.435		

Interferon Response Microglia

Two-way ANOVA	Ordinary				
Alpha	0.05				
Source of Variation	% of total variation	P value	P value summary	Significant?	
Interaction	8.438	0.2130	ns	No	
Diet	0.02917	0.9386	ns	No	
Injury	54.66	0.0088	**	Yes	
ANOVA table	SS	DF	MS	F (DFn, DFd)	P value
Interaction	40.67	1	40.67	F (1, 8) = 1.831	P=0.2130
Diet	0.1406	1	0.1406	F (1, 8) = 0.006327	P=0.9386
Injury	263.5	1	263.5	F (1, 8) = 11.86	P=0.0088
Residual	177.7	8	22.22		

Disease Associated Microglia

Two-way ANOVA	Ordinary				
Alpha	0.05				
Source of Variation	% of total variation	P value	P value summary	Significant?	
Interaction	0.01600	0.8514	ns	No	
Diet	0.2044	0.5088	ns	No	
Injury	96.36	<0.0001	****	Yes	
ANOVA table	SS	DF	MS	F (DFn, DFd)	P value
Interaction	1.279	1	1.279	F (1, 8) = 0.03743	P=0.8514
Diet	16.34	1	16.34	F (1, 8) = 0.4783	P=0.5088
Injury	7702	1	7702	F (1, 8) = 225.4	P<0.0001
Residual	273.3	8	34.17		

Pro-inflammatory-like Microglia

Two-way ANOVA	Ordinary				
Alpha	0.05				
Source of Variation	% of total variation	P value	P value summary	Significant?	
Interaction	2.816	0.3363	ns	No	
Diet	0.5815	0.6544	ns	No	
Injury	75.07	0.0007	***	Yes	
ANOVA table	SS	DF	MS	F (DFn, DFd)	P value
Interaction	13.64	1	13.64	F (1, 8) = 1.046	P=0.3363
Diet	2.818	1	2.818	F (1, 8) = 0.2161	P=0.6544
Injury	363.7	1	363.7	F (1, 8) = 27.90	P=0.0007
Residual	104.3	8	13.04		

Microglia Markers

Two-way ANOVA	Ordinary				
Alpha	0.05				
Source of Variation	% of total variation	P value	P value summary	Significant?	
Interaction	2.343	0.2989	ns	No	
Diet	17.07	0.0171	*	Yes	
Injury	65.39	0.0004	***	Yes	
ANOVA table	SS	DF	MS	F (DFn, DFd)	P value
Interaction	6.990	1	6.990	F (1, 8) = 1.234	P=0.2989
Diet	50.92	1	50.92	F (1, 8) = 8.987	P=0.0171
Injury	195.0	1	195.0	F (1, 8) = 34.43	P=0.0004
Residual	45.32	8	5.665		

Canonical genes (upregulated)

Source of Variation	% of total variation	P value	P value summary	Significant?
Injury	73.34	<0.0001	****	Yes
Diet	2.919	0.0055	**	Yes
Injury x Diet	2.919	0.0055	**	Yes
Pathway x Injury	5.874			
Pathway x Diet	4.540			
Pathway	5.874			
ANOVA table	SS	DF	MS	F (DFn, DFd)
Injury	57.09	1	57.09	F (1, 16) = 199.8
Diet	2.272	1	2.272	F (1, 16) = 10.29
Injury x Diet	2.272	1	2.272	F (1, 16) = 10.29
Pathway x Injury	4.573	16	0.2858	
Pathway x Diet	3.534	16	0.2209	
Pathway	4.573	16	0.2858	
Residual	3.534	16	0.2209	

Canonical genes (downregulated)

Source of Variation	% of total variation	P value	P value summary	Significant?	
Injury	97.29	<0.0001	****	Yes	
Diet	0.1164	0.3369	ns	No	
Injury x Diet	0.1164	0.3369	ns	No	
Pathway x Injury	0.8481				
Pathway x Diet	0.3920				
Pathway	0.8481				
ANOVA table	SS	DF	MS	F (DFn, DFd)	P value
Injury	24.19	1	24.19	F (1, 4) = 458.9	P<0.0001
Diet	0.02896	1	0.02896	F (1, 4) = 1.188	P=0.3369
Injury x Diet	0.02896	1	0.02896	F (1, 4) = 1.188	P=0.3369
Pathway x Injury	0.2109	4	0.05272		
Pathway x Diet	0.09747	4	0.02437		
Pathway	0.2109	4	0.05272		
Residual	0.09747	4	0.02437		

C Flow Through – Fig. 10

Flowthrough Neurons

Two-way ANOVA	Ordinary				
Alpha	0.05				
Source of Variation	% of total variation	P value	P value summary	Significant?	
Interaction	17.35	0.0181	*	Yes	
Diet	5.702	0.1278	ns	No	
Injury	61.15	0.0005	***	Yes	
ANOVA table	SS	DF	MS	F (DFn, DFd)	P value
Interaction	194.4	1	194.4	F (1, 8) = 8.781	P=0.0181
Diet	63.88	1	63.88	F (1, 8) = 2.886	P=0.1278
Injury	685.1	1	685.1	F (1, 8) = 30.95	P=0.0005
Residual	177.1	8	22.13		

Flowthrough Oligodendrocytes

Two-way ANOVA	Ordinary				
Alpha	0.05				
Source of Variation	% of total variation	P value	P value summary	Significant?	
Interaction	13.26	0.0485	*	Yes	
Diet	9.273	0.0877	ns	No	
Injury	57.85	0.0013	**	Yes	
ANOVA table	SS	DF	MS	F (DFn, DFd)	P value
Interaction	10.47	1	10.47	F (1, 8) = 5.410	P=0.0485
Diet	7.322	1	7.322	F (1, 8) = 3.782	P=0.0877
Injury	45.68	1	45.68	F (1, 8) = 23.60	P=0.0013
Residual	15.49	8	1.936		

Flowthrough Astrocytes

Two-way ANOVA	Ordinary				
Alpha	0.05				
Source of Variation	% of total variation	P value	P value summary	Significant?	
Interaction	12.83	0.0777	ns	No	
Diet	3.150	0.3455	ns	No	
Injury	58.93	0.0025	**	Yes	
ANOVA table	SS	DF	MS	F (DFn, DFd)	P value
Interaction	95.47	1	95.47	F (1, 8) = 4.091	P=0.0777
Diet	23.44	1	23.44	F (1, 8) = 1.005	P=0.3455
Injury	438.6	1	438.6	F (1, 8) = 18.79	P=0.0025
Residual	186.7	8	23.34		

Figure 16-Supplemental
